# A scoping review and guide for in vitro healthy human knee joint laxity

**DOI:** 10.3389/fbioe.2026.1741003

**Published:** 2026-03-18

**Authors:** Bo Eitel Seiferheld, Martin Vorup Lindvald, Ilias Theodorakos, Brett Michael Musolf, Morten Bilde Simonsen, Michael Skipper Andersen, Mohammadjavad (Matin) Einafshar

**Affiliations:** 1 Department of Materials and Production, Aalborg University, Aalborg, Denmark; 2 Center for Mathematical Modeling of Knee Osteoarthritis, Department of Materials and Production, Aalborg University, Aalborg, Denmark

**Keywords:** anterior-posterior translation, heterogeneity, internal-external rotation, knee flexion angle, knee laxity, study recommendation, varus-valgus rotation

## Abstract

**Introduction:**

Knee laxity is a well-studied concept with a vast repository of information in the literature. However, an often-overlooked challenge arises from the diverse methodological heterogeneity, making inter-study comparisons and overall knowledge of knee laxity confounded. Therefore, this review aimed to comprehensively summarise *in vitro* data on the intact knee laxity to discuss and highlight experimental trends to use the current methodological insights to establish a foundation for standardised testing guidelines.

**Materials and Methods:**

A systematic search on PubMed, Embase, and Web of Science was conducted, spanning all publications up to 30 October 2024. Here, studies providing quantitative data on intact, primary or secondary, knee joint laxity (i.e., anterior-posterior, internal-external and varus-valgus) were synthesised together with their methodological procedures. Data were grouped based on loading intervals (i.e., 88–100 N, 130–134 N, 5 Nm and 10 Nm) and knee flexion angles (i.e., 0°, 15°, 30°, 45°, 60°, 90°, and 120°), based on the most available data.

**Results:**

A total of 161 studies comprising 1741 intact knee specimens were included. Anterior-posterior laxity was the most frequently reported, followed by internal-external and varus-valgus directions. Despite comparable experimental setups, substantial variability was observed in grouped data laxity values due to differences in coordinate system definition and kinematic constraints. In fact, specimen preparation, demographics and intactness were frequently incomplete or missing, limiting confidence in the validity of reported intact knee laxity measurements.

**Discussion and Conclusions:**

Due to limited field coherence and methodological transparency, guidelines are needed for laxity reporting in the future. Thus, the synthesised information from all the included articles was used to formulate foundational guidelines for standardised testing and reporting of knee laxity in the future. These guidelines cover specimen reporting, specimen knee intactness check, laxity reporting, experimental testing and measuring conditions to enable result comparisons and future meta-analysis.

## 1 Introduction

In the biomechanical literature, the terms knee joint instability and laxity are often used interchangeably to describe a pathological deviation from the intact knee joint function (Zlotnicki et al., 2016). However, from a biomechanical standpoint, they represent two distinct concepts. Knee laxity is defined as the passive movement of the joint (i.e., translation and rotation), within the constraints of ligaments, capsule and cartilage when an external force or moment is applied in a state of complete muscular relaxation (Cross, 1996; Küpper et al., 2007). In contrast, knee joint instability refers to functional measurements and is characterised by a subjective sensation of buckling, shifting, or lack of confidence in the knee maintaining stability (Zlotnicki et al., 2016).

The human knee joint is susceptible to a variety of pathologies, ranging from degenerative changes such as osteoarthritis (OA) (Glyn-Jones et al., 2015; Sharma, 2021) to traumatic injuries like anterior cruciate ligament (ACL) tears (Hughston et al., 1976; Bram et al., 2021) and meniscal damage (Fox et al., 2015), which can lead to secondary post-traumatic arthritis (Dilley et al., 2023; Luc et al., 2014). Fundamentally, these conditions alter the inherent laxity, leading to chronic pain (Sharma, 2021; Bernad-Pineda et al., 2014; Filbay et al., 2015), and measurable changes in lower-limb biomechanics, including decreased knee flexion angle and altered knee adduction and flexion moment in symptomatic patients during gait (Moore et al., 2020; Astephen et al., 2008), along with a subjective sense of instability (Kavchak et al., 2012; Roberts et al., 2007) and reduced quality-of-life (Bernad-Pineda et al., 2014; Filbay et al., 2015). Consequently, people with advanced joint degeneration or significant ligamentous injury often face functional limitations that require surgical interventions, such as knee arthroplasty (Li et al., 2019) or ligament reconstruction (Woo et al., 2006), to relieve symptoms. Various surgical approaches may be used (Dossett et al., 2012; Oussedik et al., 2020; Chambat et al., 2013; Gerami et al., 2022), but the shared goal is to restore healthy knee motion and laxity. Establishing normative laxity values for the healthy knee would provide a fundamental reference necessary to identify pathological deviations and define objective targets for functional restoration. Furthermore, laxity data may serve as critical clinical guidelines for preoperative surgical planning, the validation of predictive computational simulations, and the objective assessment of modified joint laxity during post-operative follow-ups. Despite surgical advancements, a significant proportion of people remain dissatisfied with their surgical outcome, reporting persistent issues such as pain, instability, stiffness and swelling (Woo et al., 2006; Bourne et al., 2010). Thus, revisions are needed (Kurtz et al., 2007; Wright et al., 2012; Schwartz et al., 2020). Dissatisfaction can stem from various factors, including implant design, alignment strategy, tunnel placement, graft choice, size and tension; each potentially contributing to residual laxity post-surgery (Dossett et al., 2012; Oussedik et al., 2020; Liukkonen et al., 2022; Mariscalco et al., 2013).

A laxity profile refers to the systematic recording of passive knee joint motion, describing the flexion angle-dependent pattern of both translational and rotational motion under controlled loads. Laxity profiles have been studied using *in vivo* (Kümmerlin et al., 2022; Küpper et al., 2016, Küpper et al., 2007) and *in vitro* (Ahn et al., 2011; E Albuquerque et al., 2007; Albuquerque Ii et al., 2019; Anderson et al., 2010; Andreassen et al., 2021; Arnout et al., 2022; Athwal et al., 2016; Athwal et al., 2017; Athwal et al., 2021; Bae et al., 2008; Beel et al., 2024; Behrendt et al., 2022; Bergfeld et al., 2005; Boguszewski et al., 2015; Branch et al., 2017; Deichsel et al., 2024; Delaloye et al., 2023; DePhillipo et al., 2018; Devitt et al., 2019; Diermann et al., 2009; Fayed et al., 2022; Fujie et al., 2011; Fukubayashi et al., 1982; Gabriel et al., 2004; Gadikota et al., 2009, 2010; Galloway et al., 1996; Geeslin et al., 2018; Giffin et al., 2007; Gladnick et al., 2016; Goldsmith et al., 2013; Gollehon et al., 1987; Gupte et al., 2003; Harms et al., 2015; Harner et al., 2000; He et al., 2023; Henke et al., 2024; Herbort et al., 2010; Herbort et al., 2013; Herbort et al., 2016; Herve et al., 2025; Heyse et al., 2015; Ho et al., 2009; Hsieh and Walker, 1976; Huser et al., 2017; Imhauser et al., 2013; Inderhaug et al., 2017; Jenny et al., 2017; Jette et al., 2019; Kanamori et al., 2000; Kanamori et al., 2003; Kato et al., 2012; Kato et al., 2013; Kennedy et al., 2013, Kennedy et al., 2014; Kennedy et al., 2014b; Kilger et al., 2005; Kilger et al., 2006; Kim et al., 2010; Kondo et al., 2010; Kondo et al., 2011; Kondo et al., 2014; Küpper et al., 2022; LaPrade et al., 2010; Lenschow et al., 2006; Lertwanich et al., 2011; Li et al., 1999, 2007; Liu et al., 2014; Liu et al., 2015; Liu et al., 2020; Lo et al., 2011; Lorbach et al., 2015b, 2015a; Lord et al., 2017; Lording et al., 2017; Lorenz et al., 2016; Ma et al., 2000; Ma et al., 2003; Mae et al., 2001; Margheritini et al., 2004; Markolf et al., 1976; Markolf et al., 2008; McCarthy et al., 2013; Miura et al., 2006; Musahl et al., 2005; Musahl et al., 2007; Musahl et al., 2011; Mutnal et al., 2015; Nakamura et al., 2021; Nau et al., 2005; Ng et al., 2022; Nielsen et al., 2018; Nitri et al., 2016; Noyes et al., 2017; Noyes et al., 2018; Noyes et al., 2019; Noyes et al., 2021; Park et al., 2004; Parsons et al., 2015; Pascual-Leone et al., 2024; Pearsall et al., 1996; Pedersen et al., 2019; Petersen et al., 2007; 2008; Race and Amis, 1998; Rasmussen et al., 2016; Razu et al., 2023; Reynolds et al., 2017; Robinson et al., 2006; Roth et al., 2015; Rudy et al., 2000; Ruiz et al., 2016; Russell et al., 2014; Sakamoto et al., 2020; Sakane et al., 1999; Sandriesser et al., 2022; Sasaki et al., 2007; Sasaki et al., 2014; Sasaki et al., 2016; Schliemann et al., 2017; Sekiya et al., 2005; Sena et al., 2013; Senioris et al., 2017; Seon et al., 2010; Serbino et al., 2015; Shino et al., 1987; Sim et al., 2011; Stephen et al., 2016; Suggs et al., 2004; Sullivan et al., 1984; Suzuki et al., 2019; Tsai et al., 2010; Tsukada et al., 2012; Vogrin et al., 2000; Vrancken et al., 2016; Walker et al., 2015; Wang et al., 2014; Watanabe et al., 2004; Weimann et al., 2012; 1988; Wierer et al., 2021; Wijdicks et al., 2013b, Wijdicks et al., 2013b; Wilhelm et al., 2023; Willinger et al., 2023; Wilson et al., 2013; Winkelmeyer et al., 2021; Woo et al., 2002; Xu et al., 2011, 2022; Yagi et al., 2002; Yamamoto et al., 2004; Yoo et al., 2005; Zamarra et al., 2010; Zantop et al., 2007a; Zantop et al., 2007b; Zantop et al., 2008; Zantop et al., 2010; Özbek et al., 2023) methods. *In vivo* measurements are ideal for replicating real-world conditions, as they preserve the natural pretension and constraints of ligaments, the joint capsule, tendons and cartilage (Küpper et al., 2007). In contrast, in vitro methods offer greater experimental control, enabling accurate assessment of the knee laxity profile (Ahldén et al., 2012). Additionally, it provides a unique opportunity for intra-subject comparison of intact conditions against induced artificial ligament deficiencies and reconstruction methods that would otherwise be unethical or impractical *in vivo*. Therefore, a larger proportion of data is available to describe the laxity profile of the intact knee joint *in vitro* than *in vivo*.

Despite a vast repository of information in the literature, an often overlooked challenge arises from the diverse array of techniques and measurement devices reported, making inter-study comparisons challenging (Ahldén et al., 2012). In fact, a recent meta-analysis demonstrated substantial heterogeneity in reported knee laxity measures (Ferle et al., 2019). This variability in methodologies adds an additional layer of complexity when consolidating findings, making it challenging to draw consistent conclusions. Although recent efforts have been made to establish best-practice guidelines for conducting *in vitro* knee experiments, these focus primarily on defining the clinical question of interest and the experimental setup and measurement procedures, while offering limited direction on data reporting, specimen preparation and results comparison (Colbrunn et al., 2024). Hence, addressing this methodological diversity is essential for advancing the understanding of knee laxity. Thus, a comprehensive overview is needed to clarify how *in vitro* knee laxity testing is currently conducted and to support the development of meaningful guidelines for future research, as no consensus currently exists within the literature.

Therefore, in this scoping review, we aim to comprehensively summarise *in vitro* data on the intact knee laxity. Specifically, we (i) systematically review knee laxity profiles; (ii) discuss and highlight trends in testing techniques and protocols; and (iii) incorporate insights from the current methodologies to establish a foundation for standardised guidelines.

## 2 Materials and methods

### 2.1 Identification of studies via databases and registers

A literature search was conducted across three digital databases: PubMed, Embase, and Web of Science. The search spanned all publications within the database origin up to 30 October 2024, which served as the final search date, aiming to identify studies providing quantitative data on knee joint laxity and measurement techniques used. The full search query is available in Supplementary Material (S1). Subsequently, titles and abstracts were screened based on the following inclusion criteria: (1) joint laxity was measured from healthy intact human cadaveric knees (2) laxity was measured as passive joint movement (i.e., translation and rotation); in a state of complete muscular relaxation; (3) laxity was tested under known external stimuli (i.e., known force and moment); (4) data were reported as quantitative measure of laxity; and (5) journal articles were written in English with the full text available. Studies focusing solely on the patellofemoral joint or those involving knees with pathological conditions were excluded.

### 2.2 Identification of studies via other methods

In addition to the systematic literature review, a chain-searching process was performed to find additional studies. This involved reviewing the reference lists of included articles, examining previously published reviews, and performing targeted searches on Google Scholar. Additional studies found were, likewise, screened based on the inclusion criteria outlined in Section 2.1.

### 2.3 Title and abstract screening

Title and abstract screening were conducted with all authors blinded on Rayyan.ai (Ouzzani et al., 2016). Authors were divided into pairs, and each author individually screened their studies and categorised them as “included,” “excluded,” or “maybe.” Subsequently, each author pair held intragroup meetings to resolve potential discrepancies. If a discrepancy persisted, one author (M.S.A) reviewed the full text to make a final decision. Lastly, the full text was assessed and analysed for data extraction.

### 2.4 Data extraction and handling

#### 2.4.1 Kinematics

Data were extracted following a standardised form, and the complete overview of the raw data is available in Supplementary Material (S2). For each included article, mean values and corresponding standard deviations of knee laxity were digitised at the prescribed knee flexion angles reported. Data were extracted for three degrees of freedom (DOF; anterior and posterior translation, varus and valgus angulation, and internal and external rotation). Laxity data were divided into primary and secondary laxity as well as stratified by single and multiple load conditions. Primary laxity refers to joint movement in the same direction as one of the applied loads, while secondary laxity refers to joint movements that occur in directions different from the applied load. Analyses were restricted to primary laxity measured under single load protocols, meaning only primary laxity where the singular applied load is in the same direction as the measured direction.

In cases where laxity data were published in graphical format, data were manually digitised using the Engauge Digitizer software (Mitchell et al., 2020) (version 12.2.1). The discretised tool was used to enhance visibility, and axis points were marked to define the coordinate system for the two-dimensional data in the software. From here, the curve point tool was used to mark and extract the laxity data points. To synthesise the data and account for both intra-study variability and inter-study differences, a weighted approach based on sample size was used to compute an overall mean ( $\hat{\mu }$ ) and standard deviation ( $\hat{\sigma }$ ) for each knee flexion angle (Equations 1, 2) (Wan et al., 2014).

$$\hat{\mu }=\frac{\sum_{i=1}^{k}n_{i}{\hat{\mu }}_{i}}{\sum_{i=1}^{k}n_{i}}$$ (1)

$$\hat{\sigma }=\sqrt{\frac{\sum_{i=1}^{k}\left(n_{i}-1\right){\hat{\sigma }}_{i}^{2}+n_{i}{\left({\hat{\mu }}_{i}-\hat{\mu }\right)}^{2}}{\left(\sum_{i=1}^{k}n_{i}\right)-1}}$$ (2)

Where *k* represents the total number of included studies, *n*_*i*_ is the sample size in the *i*th study, ${\hat{\mu }}_{i}$ is the mean value of the *i*th study, ${\hat{\sigma }}_{i}^{2}$ is the variance in the *i*th study.

Due to a high variability in applied forces and moments, with some values supported by many knee samples and others by very few, the data were grouped in quantified loading intervals to ensure sufficient data representation. Force data were grouped into knee samples tested at 88–100 N and 130–134 N, while moment data were grouped into 5 Nm and 10 Nm, as these ranges contained most of the reported values (Supplementary Material S3). An equivalent procedure was applied for specific knee flexion angles, where 0°, 15°, 30°, 45°, 60°, 90°, and 120° were used (Supplementary Material S3).

#### 2.4.2 Specimen characteristics, preparation and intactness

Selected study characteristics related to the specimens were extracted. Specifically, sample size, age, gender, and body mass index (BMI).

Moreover, the specimen preparation procedure of the knee was evaluated and categorised based on the presence and condition of ligaments, muscles, skin and muscle tension. The categories included: ligaments only (L), ligaments with partial muscles (L/P), ligaments with intact muscle (L/I), ligaments with partial muscle and skin (L/P/S), ligaments with intact muscle and skin (L/I/S) and ligaments with intact skin, muscle and applied tensions (L/S/T).

Lastly, an assessment metric was developed to assess knee intactness based on (1) the information provided to determine if the knee was indeed intact, (2) the degree of reproducibility of the methods used to determine the intactness and (3) the degree of validity of the methodology described and used to determine the state of intactness. The three dimensions were scored on an increasing scale from 0 to 5, where (0) was no description and (5) was a complete description. For example, some assessed the integrity of knee specimens through visual inspection for scars or evidence of prior surgery or injury (Albuquerque Ii et al., 2019). This approach was considered weak, as it relied only on superficial inspection and lacked methodological rigor. More rigorous methods included excluding specimens with prior ligament injury or surgery, evaluating arthritis and ligament injury using fluoroscopy and manual laxity tests, inspecting intra-articular structures via arthroscopy, and excluding knees with cartilage injury above Outerbridge classification II or meniscal tears (Seon et al., 2010).

#### 2.4.3 Measuring, capturing device and coordinate system

Study characteristics related to the measurement setup were extracted. Specifically, the testing apparatus: robotic arm (RA), universal testing machine (TM), clinical device (CD), or miscellaneous (MISC) and the capturing system: computer-assisted surgery and kinematics (CAS), electromagnetic tracking (ETD), stereoradiography (SR), magnetic resonance imaging (MRI), kinematics from the load applicator (LA), optical system (OPT), clinical device (CD), or miscellaneous (MISC). Furthermore, the coordinate system defined for each study was categorized as: not available (N/A), custom defined coordinate system (Custom), coordinate system of the International Society of Biomechanics (ISB) proposed by Grood and Suntay (1983).

## 3 Results

**FIGURE 1:**
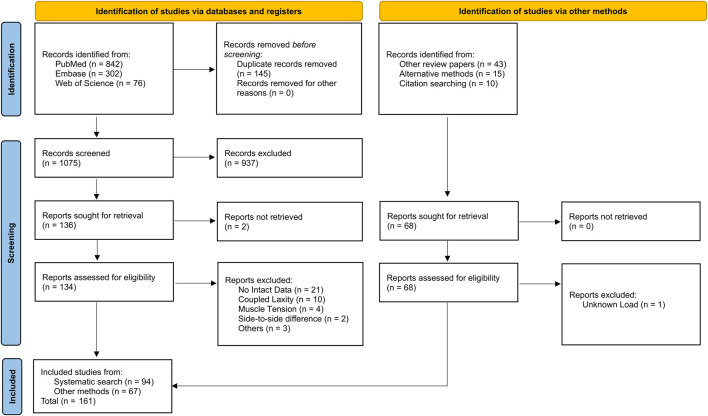
PRISMA flow diagram of study selection, showing records identified, screened, assessed for eligibility, and included, with reasons for exclusions (Page et al., 2021).

The systematic literature search identified 1,220 studies in PubMed, Embase and Web of Science electronic databases. Following the addition of relevant studies obtained through other methods (n = 68), duplicate removal, title and abstract-based screening, and exclusion of non-eligible studies, 161 studies were included (Figure 1). Of the excluded full-text articles (n = 41), 21 were removed as no intact baseline was reported, 10 because laxity was reported as a coupled laxity measure, four studied used muscle tension during testing, two studies reported side-to-side differences, three studies were excluded for other reasons and one study had unknown loads. Here, no intact data indicates that laxity was only reported after an experimental condition was applied (e.g., after ligament transection) without reporting a healthy baseline; coupled laxity denotes studies that reported a combined laxity measure of two or more directions rather than separate directional values (e.g., combined anterior-posterior laxity); muscle tension refers to protocols in which simulated muscle loading was applied during laxity testing; side-to-side differences describes outcomes reported solely as differences between limbs or conditions without the underlying absolute laxity values.

Primary and secondary laxity of 1741 knees were evaluated at flexion angles from −5 to 135°. The laxity was measured by applying varying loads of 5–500 N for the anterior (121 studies) and 5–200 N for the posterior directions (54 studies), 2 to 29 Nm for varus rotation (31 studies), 3 to 15 Nm for valgus rotation (39 studies), 1.5 to 14 Nm for internal rotation (84 studies), and 1.5 to 10 Nm for external rotation (54 studies) (Table 1).

**TABLE 1:** Summary of experimental testing conditions of all articles (n = 161).

Lead author (Year)	Sample size	Testing class	Capturing class	Constrained	Applied force & moment						Measurement direction						Knee flexion range
					ANT (N)	POST (N)	INT (Nm)	EXT (Nm)	VALG (Nm)	VAR (Nm)	ANT	POST	INT	EXT	VALG	VAR	
Ahn et al. (2011)	10	TM	OPT	Yes	134	​	5	​	10	​	Y^P^	​	Y^PM^	​	Y^S^	​	0–90
Albuquerque et al. (2007)	20	TM	LA	N/A	100	​	​	​	​	​	Y^P^	​	​	​	​	​	0–90
Albuquerque Ii et al. (2019)	8	MISC	OPT	No	​	100	​	​	​	​	​	Y^P^	​	​	​	​	10–90
Anderson et al. (2010)	12	MISC	ETD	No	88	​	5	​	10	​	Y^PM^, Y^P^	​	Y^P^	​	Y^P^	​	0–90
Andreassen et al. (2021)	2	MISC	SR	No	100	​	3	​	​	​	Y^PM^	​	Y^PM^	​	​	​	30–30
Arnout et al. (2022)	14	MISC	OPT	No	100	100	1.5	1.5	7.5	7.5	Y^P^	Y^P^	Y^P^	Y^P^	Y^P^	Y^P^	0–120
Athwal et al. (2016)	8	RA	LA	No	90	90	5	5	8	8	Y^P^	Y^P^	Y^P^	Y^P^	Y^P^	Y^P^	5–80
Athwal et al. (2017)	9	RA	LA	No	90	90	5	5	8	8	Y^P^	Y^P^	Y^P^	Y^P^	Y^P^	Y^P^	0–90
Athwal et al. (2021)	10	RA	LA	No	90	90	5	5	8	8	Y^P^	Y^P^	Y^P^	Y^P^	Y^P^	Y^P^	0–90
Bae et al. (2008)	14	MISC	MISC	Yes	​	​	​	6	​	​	​	​	​	Y^P^	​	​	30–30
Beel et al. (2024)	16	RA	OPT	No	89	​	4	4	8	​	Y^PM^	​	Y^P^	Y^P^	Y^P^	​	0–90
Behrendt et al. (2022)	8	RA	LA	No	134	​	​	5	10	​	Y^PM^	​	​	Y^P^	Y^P^	​	0–90
Bergfeld et al. (2005)	8	TM	LA	Yes	​	100	​	​	​	​	​	Y^P^	​	​	​	​	10–90
Boguszewski et al. (2015)	25	RA	LA	Yes	134	134	5	5	10	10	Y^P^	Y^P^	Y^P^	Y^P^	Y^P^	Y^P^	0–50
Branch et al. (2017)	1	MISC	LA	N/A	134	​	​	​	6	​	​	Y^S^	​	​	Y^P^	​	30–30
Deichsel et al. (2024)	8	RA	LA	Yes	89	​	​	5	8	​	Y^PM^, Y^P^	​	​	Y^P^	Y^P^	​	0–90
Delaloye et al. (2023)	12	RA	LA	N/A	134	​	5	​	​	​	Y^P^	​	Y^P^	​	​	​	0–90
DePhillipo et al. (2018)	24	RA	LA	N/A	88	​	​	​	​	​	Y^P^	​	​	​	​	​	30–90
Devitt et al. (2019)	12	RA	LA	Yes	90	​	4, 5	​	8	​	Y^S^, Y^P^	​	Y^P^	​	​	​	0–90
Diermann et al. (2009)	7	RA	LA	No	134	​	4	​	10	​	Y^S^, Y^P^	​	Y^PM^	​	​	​	0–90
Fayed et al. (2022)	15	RA	MISC	No	89	89	5	5	7	​	Y^S^, Y^P^	Y^P^	​	Y^P^	​	​	0–90
Fujie et al. (2011)	5	RA	LA	Yes	120	120	​	​	​	​	Y^S^	​	​	​	​	​	0–90
Fukubayashi et al. (1982)	9	TM	LA	Yes	50, 100	50, 100	​	​	​	​	Y^P^	Y^P^	Y^S^	Y^S^	​	​	0–90
Gabriel et al. (2004)	10	RA	LA	N/A	134	​	5	​	10	5	Y^S^, Y^P^	​	Y^S^	​	Y^S^	​	0–90
Gadikota et al. (2009)	8	RA	LA	N/A	130	​	5	​	5	​	Y^S^, Y^P^	​	Y^PM^	​	​	​	0–90
Gadikota et al. (2010)	9	RA	LA	N/A	134	​	5	​	10	​	Y^S^, Y^P^	​	Y^PM^	Y^S^	​	​	0–90
Galloway et al. (1996)	12	MISC	LA	No	​	100	​	​	​	​	​	Y^P^	​	​	​	​	0–105
Geeslin et al. (2018)	18	RA	LA	No	88	​	5	​	10	​	Y^S^, Y^P^	​	Y^PM^, Y^P^	​	​	​	0–90
Giffin et al. (2007)	10	RA	LA	Yes	134	134	​	​	​	​	Y^S^, Y^P^	Y^P^	​	​	​	​	0–120
Gladnick et al. (2016)	20	RA	MISC	Yes	​	​	​	​	4	4	Y^S^	​	Y^S^	​	Y^P^	Y^P^	0–90
Goldsmith et al. (2013)	18	RA	LA	Yes	88	​	5	5	10	10	Y^S^, Y^P^	​	Y^P^	Y^P^	Y^P^	Y^P^	0–90
Gollehon et al. (1987)	17	TM	LA	Yes	100	100	4.5, 5.4	4.5	10	10	Y^S^, Y^P^	Y^S^, Y^P^	Y^S^, Y^P^	Y^S^, Y^P^	Y^P^	Y^P^	0–90
Gupte et al. (2003)	8	TM	LA	No	100	100	5	5	​	​	Y^P^	Y^P^	Y^P^	Y^P^	​	​	0–120
Harms et al. (2015)	10	MISC	LA	No	35, 100	​	1, 5	​	7	​	Y^PM^, Y^P^	​	Y^PM^, Y^P^	​	​	​	25–25
Harner et al. (2000)	10	RA	LA	No	​	134	​	​	​	​	​	Y^P^	​	​	​	​	0–120
He et al. (2023)	14	RA	LA	Yes	89	​	5	5	7	​	Y^S^, Y^P^	​	Y^P^	Y^P^	​	​	0–90
Henke et al. (2024)	1	RA	LA	Yes	40	40	2.5	2.5	5	5	Y^P^	Y^P^	Y^P^	Y^P^	Y^P^	Y^P^	30–90
Herbort et al. (2010)	9	RA	LA	No	134	​	4	​	10	​	Y^S^, Y^P^	​	​	​	​	​	0–90
Herbort et al. (2013)	9	RA	LA	Yes	134	​	4	​	10	​	Y^S^, Y^P^	​	​	​	​	​	0–90
Herbort et al. (2016)	10	RA	LA	Yes	143	​	5	​	10	​	Y^S^, Y^P^	​	​	​	​	​	0–90
Herve et al. (2025)	7	RA	LA	No	134	134	​	​	10	10	Y^P^	Y^P^	​	​	Y^P^	Y^P^	0–90
Heyse et al. (2015)	6	RA	LA	Yes	200	​	​	​	10	10	​	​	​	​	Y^PM^	Y^PM^	15–90
Ho et al. (2009)	16	MISC	OPT	No	133	​	10	10	​	​	Y^S^, Y^P^	Y^S^	Y^S^, Y^P^	Y^P^	​	​	30–60
Hsieh and Walker (1976)	4	TM	MISC	Yes	98	​	​	4.9	​	​	Y^P^	​	​	Y^P^	​	​	0–30
Huser et al. (2017)	19	MISC	LA	No	100	​	5	​	7	​	Y^P^	​	Y^P^	​	​	​	25–90
Imhauser et al. (2013)	11	RA	OPT	No	134	​	4	​	​	8	Y^S^, Y^P^	​	Y^PM^	​	​	Y^S^	0–90
Inderhaug et al. (2017)	12	MISC	OPT	No	90	​	5	​	​	​	Y^P^	​	Y^P^	​	​	​	0–90
Jenny et al. (2017)	10	CD	CD	No	200, 250, 150, 134	​	​	​	​	​	Y^P^	​	​	​	​	​	20–20
Jette et al. (2019)	12	TM	LA	No	90	​	7	​	​	​	Y^P^	​	Y^P^	​	​	​	0–90
Kanamori et al. (2000)	12	RA	LA	No	​	​	10	​	10	​	Y^S^, Y^P^	​	Y^PM^, Y^P^	​	​	​	0–90
Kanamori et al. (2003)	10	RA	LA	No	​	134	​	10	​	​	​	Y^P^	​	Y^P^	​	​	0–90
Kato et al. (2012)	18	RA	LA	No	89	​	5	​	7	​	Y^S^, Y^P^	​	​	​	​	​	0–60
Kato et al. (2013)	16	RA	LA	No	89	​	5	​	7	​	Y^S^, Y^P^	​	​	​	​	​	0–60
Kennedy et al. (2013)	20	RA	OPT	Yes	​	100, 134	5	5	10	10	​	Y^PM^, Y^P^	Y^P^	Y^PM^, Y^P^	Y^P^	Y^P^	0–120
Kennedy et al. (2014a)	9	RA	OPT	Yes	​	134	5	​	10	​	​	Y^P^	Y^P^	​	Y^P^	​	0–120
Kennedy et al. (2014b)	9	RA	LA	Yes	​	134	5	5	10	​	​	Y^P^	Y^P^	Y^P^	Y^P^	​	0–120
Kilger et al. (2005)	8	RA	LA	Yes	134	​	5	​	10	​	Y^S^, Y^P^	​	Y^PM^	​	​	​	0–90
Kilger et al. (2006)	10	RA	LA	Yes	134	​	5	​	10	​	Y^S^, Y^P^	​	Y^PM^	​	​	​	15–90
Kim et al. (2010)	36	MISC	MISC	No	​	​	​	5	​	3	​	​	​	Y^P^	​	Y^P^	0–90
Kim et al. (2015)	10	RA	LA	No	89	​	5	​	7	​	Y^S^, Y^P^	​	​	​	​	​	0–90
Kondo et al. (2010)	8	MISC	OPT	No	90	​	5	​	​	​	Y^P^	​	Y^P^	​	​	​	0–110
Kondo et al. (2011)	8	MISC	OPT	No	90	90	5	5	​	​	Y^P^	Y^P^	Y^P^	Y^P^	​	​	0–110
Kondo et al. (2014)	14	MISC	OPT	No	90	90	5	5	​	​	Y^P^	Y^P^	Y^P^	Y^P^	​	​	0–110
Küpper et al. (2022)	4	MISC	MRI	Yes	40, 89, 139	​	​	​	​	​	Y^P^	​	​	​	​	​	30–30
LaPrade et al. (2010)	11	MISC	OPT	No	88	88	5	5	​	10	Y^P^	Y^P^	Y^P^	Y^P^	​	Y^P^	0–90
Lenschow et al. (2006)	10	RA	LA	Yes	​	134	​	​	​	​	​	Y^P^	​	​	​	​	0–90
Lertwanich et al. (2011)	10	RA	LA	Yes	89	​	​	​	​	​	Y^P^	​	​	​	​	​	0–90
Li et al. (1999)	1	RA	LA	Yes	100, 40, 80, 20, 60	100, 40, 80, 20, 60	​	​	​	​	Y^P^	Y^P^	​	​	​	​	0–30
Li et al. (2007)	9	RA	LA	N/A	130	​	​	​	​	​	​	​	​	​	​	Y^S^	0–90
Liu et al. (2014)	12	RA	LA	Yes	​	​	5	5	​	10	​	​	Y^P^	Y^P^	​	Y^P^	0–120
Liu et al. (2015)	6	RA	LA	No	​	​	5	5	​	​	​	​	Y^P^	Y^P^	​	​	15–120
Liu et al. (2020)	12	RA	LA	Yes	​	134	5	5	​	10	​	Y^P^	Y^P^	Y^P^	​	Y^P^	0–90
Lo et al. (2011)	14	RA	LA	Yes	130	130	5	5	5	5	Y^P^	Y^P^	Y^P^	Y^P^	Y^P^	Y^P^	0–90
Lorbach et al. (2015a)	18	RA	LA	Yes	134	​	4	​	10	​	Y^S^, Y^P^	​	​	​	​	​	0–90
Lorbach et al. (2015b)	12	RA	LA	Yes	134	​	4	​	10	​	Y^S^, Y^P^	​	​	​	​	​	0–90
Lord et al. (2017)	9	RA	LA	Yes	90	​	4, 5	5	8	​	Y^S^, Y^P^	​	Y^PM^, Y^P^	Y^P^	​	​	0–90
Lording et al. (2017)	12	RA	ETD	Yes	200	200	5	5	14	14	Y^S^	Y^S^	Y^S^	Y^S^, Y^P^	Y^P^, Y^S^	Y^S^	30–30
Lorenz et al. (2016)	10	RA	LA	Yes	80	​	4	4	​	​	Y^P^	​	Y^P^	Y^P^	​	​	20–90
Ma et al. (2000)	36	RA	LA	Yes	134	134	​	​	​	​	Y^P^	Y^P^	​	​	​	​	0–120
Ma et al. (2003)	10	RA	LA	Yes	​	134	​	​	​	​	​	Y^P^	​	​	​	​	0–120
Mae et al. (2001)	7	RA	LA	Yes	100	​	​	​	​	​	Y^P^	​	​	​	​	​	0–90
Margheritini et al. (2004)	10	RA	LA	Yes	​	134	​	​	​	​	​	Y^P^	​	​	​	​	0–120
Markolf et al. (1976)	35	MISC	MISC	Yes	100	​	8	​	​	29	Y^P^	​	Y^P^	​	​	Y^P^	0–135
Markolf et al. (2008)	12	MISC	MISC	No	100	​	5	​	​	​	Y^P^	​	Y^P^	​	​	​	0–120
McCarthy et al. (2013)	10	RA	LA	No	132	​	4	​	8	​	Y^S^, Y^P^	​	Y^P,^ Y^PM^	​	​	​	0–90
Miura et al. (2006)	10	RA	LA	Yes	134	​	5	​	10	​	Y^S^, Y^P^	​	Y^PM^	​	​	​	0–90
Musahl et al. (2005)	10	RA	LA	Yes	134	​	5	​	10	​	Y^S^, Y^P^	​	​	​	​	​	0–90
Musahl et al. (2007)	4	MISC	ETD	No	​	​	6	6	​	​	​	​	Y^P^	Y^P^	​	​	0–90
Musahl et al. (2011)	10	MISC	CAS	No	68	​	​	​	​	​	Y^P^	​	​	​	​	​	20–20
Mutnal et al. (2015)	8	RA	MISC	Yes	​	150	​	​	​	​	​	​	​	Y^S^	​	​	0–120
Nakamura et al. (2021)	14	RA	LA	Yes	89	​	5	5	10	​	Y^S^, Y^P^	​	Y^P^	Y^P^	​	​	15–90
Nau et al. (2005)	9	MISC	ETD	Yes	​	100	​	5	​	​	​	Y^P^	​	Y^P^	​	​	30–90
Ng et al. (2022)	8	TM	ETD	Yes	150	​	​	​	​	​	Y^P^	​	​	​	​	​	20–20
Nielsen et al. (2018)	8	RA	SR	Yes	​	​	4	​	​	​	​	​	Y^P^	​	​	​	0–60
Nitri et al. (2016)	10	RA	LA	Yes	​	​	5	​	10	​	Y^S^	​	Y^PM^	​	​	​	0–60
Noyes et al. (2017)	7	MISC	LA	Yes	100	​	1, 5	​	7	​	Y^S^	​	​	​	​	​	25–25
Noyes et al. (2018)	8	RA	LA	Yes	​	​	5	​	​	​	​	​	Y^P^	​	​	​	15–90
Noyes et al. (2019)	7	RA	LA	Yes	100	​	​	​	​	​	Y^P^	​	​	​	​	​	25–25
Noyes et al. (2021)	15	RA	LA	Yes	100	​	1, 5	​	​	​	Y^P^	​	​	​	​	​	25–25
Park et al. (2004)	6	RA	LA	Yes	​	130	​	​	​	​	​	Y^P^	​	​	​	​	0–120
Parsons et al. (2015)	12	RA	MISC	N/A	134	​	5	​	​	​	Y^P^	​	Y^P^	​	​	​	0–90
Pascual-Leone et al. (2024)	8	RA	LA	N/A	​	​	5	5	​	​	​	​	Y^P^	Y^P^	​	​	0–0
Pearsall et al. (1996)	7	MISC	MISC	Yes	​	100	​	​	​	​	​	Y^P^	​	​	​	​	0–90
Pedersen et al. (2019)	4	MISC	SR	No	67, 134	67, 134	3, 6	3	​	​	Y^PM^,Y^P^	Y^PM^,Y^P^	Y^PM^, Y^P^	Y^PM^, Y^P^	Y^S^	Y^S^	30–30
Petersen et al. (2007)	10	RA	LA	Yes	134	​	5	​	10	​	Y^S^, Y^P^	​	​	​	​	​	0–90
Petersen et al. (2008)	10	RA	LA	Yes	​	134	5	​	10	​	​	Y^S^, Y^P^	​	​	​	​	0–90
Race and Amis (1998)	8	TM	LA	Yes	​	100	​	​	​	​	Y^P^	Y^P^	​	​	​	​	0–130
Rasmussen et al. (2016)	10	RA	LA	Yes	88	​	5	​	10	​	Y^PM^	​	Y^PM^	​	​	​	0–120
Razu et al. (2023)	2	RA	LA	Yes	30	​	2	​	8	​	Y^PM^	​	Y^PM^	​	Y^PM^	​	30–30
Reynolds et al. (2017)	5	MISC	MISC	Yes	100	100	2	​	​	​	Y^P^	Y^P^	​	​	​	​	15–15
Robinson et al. (2006)	18	TM	MISC	No	150	150	5	5	5	​	Y^P^	Y^P^	Y^P^	Y^P^	Y^P^	​	0–90
Roth et al. (2015)	10	MISC	LA	Yes	45	45	3	3	5	5	Y^P^	Y^P^	Y^P^	Y^P^	Y^P^	Y^P^	0–90
Rudy et al. (2000)	10	RA	LA	Yes	100	​	​	​	​	​	Y^P^	​	Y^S^	​	​	​	0–120
Ruiz et al. (2016)	12	CD	CD	Yes	​	​	5	​	​	​	​	​	Y^P^	​	​	​	30–30
Russell et al. (2014)	12	MISC	CAS	Yes	​	​	​	​	15	15	​	​	​	​	Y^P^	Y^P^	0–0
Sakamoto et al. (2020)	10	TM	LA	Yes	100	​	​	​	​	​	Y^P^	​	​	​	​	​	30–90
Sakane et al. (1999)	10	RA	LA	Yes	110	110	​	​	​	​	Y^P^	Y^P^	​	​	​	​	0–90
Sandriesser et al. (2022)	6	TM	LA	Yes	89	​	​	​	​	​	Y^P^	​	​	​	​	​	0–90
Sasaki et al. (2007)	20	TM	LA	Yes	​	100	​	​	​	​	​	Y^P^	​	​	​	​	90–90
Sasaki et al. (2014)	10	RA	LA	No	134	​	5	​	10	​	Y^S^, Y^P^	​	Y^PM^	​	​	​	0–90
Sasaki et al. (2016)	12	RA	LA	No	89	​	5	​	7	​	Y^S^, Y^P^	​	​	​	​	​	0–90
Schliemann et al. (2017)	8	RA	LA	N/A	134	​	4	​	10	​	Y^S^, Y^P^	​	​	​	​	​	0–90
Sekiya et al. (2005)	10	RA	LA	No	​	134	​	5	​	​	​	Y^S^, Y^P^	​	Y^S^	​	​	0–90
Sena et al. (2013)	6	MISC	OPT	No	​	​	2.5	​	5.5	​	Y^S^	​	Y^PM^	​	​	​	60–60
Senioris et al. (2017)	14	CD	CD	No	250, 134	​	​	​	​	​	Y^P^	​	​	​	​	​	30–30
Seon et al. (2010)	10	RA	LA	No	134	​	5	​	10	​	Y^S^, Y^P^	​	Y^PM^	​	​	​	0–90
Serbino et al. (2015)	10	MISC	OPT	Yes	​	​	​	2, 5	​	2, 5	​	​	​	Y^P^	​	Y^P^	0–90
Shino et al. (1987)	3	MISC	MISC	No	200	200	​	​	​	​	Y^P^	Y^P^	​	​	​	​	20–20
Sim et al. (2011)	8	RA	LA	No	134	​	5	​	10	​	Y^S^, Y^P^	​	​	​	​	​	0–90
Stephen et al. (2016)	9	RA	OPT	Yes	90	​	​	​	​	​	Y^P^	​	​	​	​	​	0–100
Suggs et al. (2004)	7	RA	LA	Yes	130	130	​	​	​	​	Y^P^	Y^P^	​	​	​	​	0–120
Sullivan et al. (1984)	10	MISC	MISC	Yes	100	100	​	​	​	​	Y^P^	​	Y^S^	Y^S^	​	​	0–90
Suzuki et al. (2019)	11	RA	OPT	No	100	​	5	​	10	​	Y^S^, Y^P^	​	​	​	​	​	0–90
Tsai et al. (2010)	7	MISC	OPT	No	88	88	5	5	10	10	Y^S^, Y^P^	Y^P^	Y^P^	Y^P^	Y^P^	Y^P^	0–90
Tsukada et al. (2012)	8	TM	LA	Yes	​	100	​	5	​	​	​	Y^P^	​	​	​	​	0–90
Vogrin et al. (2000)	10	RA	LA	Yes	​	134	​	5	​	​	​	Y^P^	​	Y^P^	​	​	0–120
Vrancken et al. (2016)	5	MISC	SR	No	67	​	​	​	​	​	Y^P^	​	​	​	​	​	30–90
Walker et al. (2015)	10	MISC	OPT	Yes	100	100	2.5	2.5	​	​	Y^S^, Y^P^	Y^P^	Y^P^	Y^P^	​	​	-5-135
Wang et al. (2014)	11	RA	OPT	No	​	​	​	​	10, 6	10, 6	Y^S^	Y^S^	Y^S^	Y^S^	Y^P^	Y^P^	0–90
Watanabe et al. (2004)	10	RA	LA	Yes	​	​	​	​	​	10	Y^S^	​	​	Y^S^	​	​	0–90
Weimann et al. (2012)	10	RA	LA	Yes	​	134	5	​	10	​	​	Y^P^	Y^P^	​	Y^P^	​	0–90
Whiteside and Amador (1988)	7	RA	MISC	No	45	​	2	2	10	10	Y^P^	​	Y^P^	Y^P^	Y^P^	Y^P^	0–90
Wierer et al. (2021)	28	RA	OPT	No	89	​	4	4	8	​	Y^PM^	​	Y^P^	Y^PM^, Y^P^	Y^P^	​	0–90
Wijdicks et al. (2013a)	18	RA	LA	Yes	100	​	5	5	10	​	Y^PM^	​	Y^PM^, Y^P^	Y^P^	Y^P^	​	0–90
Wijdicks et al. (2013b)	18	RA	LA	Yes	​	100, 134	5	5	10	10	​	Y^PM^,Y^P^	Y^P^	Y^PM^, Y^P^	Y^P^	Y^P^	0–120
Wilhelm et al. (2023)	1	RA	LA	N/A	​	​	5	5	5	5	​	​	Y^P^	Y^P^	Y^P^	Y^P^	10–90
Willinger et al. (2023)	10	RA	LA	N/A	88	​	5	​	8	​	Y^S^, Y^P^	​	Y^PM^, Y^P^	​	​	​	0–100
Wilson et al. (2013)	10	MISC	OPT	Yes	​	​	​	​	11	10	​	​	​	​	Y^P^	Y^P^	30
Winkelmeyer et al. (2021)	10	MISC	MISC	No	147	​	​	​	​	​	Y^P^	​	Y^S^	​	Y^S^	​	90–90
Woo et al. (2002)	12	RA	LA	Yes	134	​	10	​	10	​	Y^S^, Y^P^	​	​	​	​	​	0–90
Xu et al. (2011)	7	RA	LA	Yes	89	​	5	​	7	​	Y^S^, Y^P^	​	​	​	​	​	0–90
Xu et al. (2022)	8	MISC	MISC	Yes	90, 100	​	5	​	10	​	Y^PM^,Y^P^	​	Y^P^	​	​	​	0–90
Yagi et al. (2002)	10	RA	LA	Yes	134	​	5	​	10	​	Y^S^, Y^P^	​	​	​	​	​	0–90
Yamamoto et al. (2004)	10	RA	LA	Yes	134	​	5	​	10	​	Y^S^, Y^P^	​	Y^PM^	​	​	​	0–90
Yoo et al. (2005)	8	RA	LA	No	130	​	​	​	​	​	Y^P^	​	​	​	​	​	0–90
Zamarra et al. (2010)	10	RA	LA	Yes	134	​	5	​	10	​	Y^S^, Y^P^	​	Y^PM^, Y^P^	​	​	​	0–120
Zantop et al. (2007a)	10	RA	LA	Yes	134	​	4	​	10	​	Y^S^, Y^P^	​	​	​	​	​	0–90
Zantop et al. (2007b)	10	RA	LA	No	134	​	4	​	10	​	Y^S^, Y^P^	​	Y^PM^, Y^P^	​	​	​	0–90
Zantop et al. (2008)	12	RA	LA	No	134	​	4	​	10	​	Y^S^, Y^P^	​	​	​	​	​	0–90
Zantop et al. (2010)	10	RA	LA	Yes	134	​	4	​	10	​	Y^S^, Y^P^	​	​	​	​	​	0–90
Özbek et al. (2023)	10	RA	LA	N/A	89	​	5	​	7	​	Y^S^, Y^P^	​	Y^P^	​	​	​	0–90

Testing class: Robot arm (RA), universal testing machines (TM), clinical device (CD) or miscellaneous (MISC). Capturing class: Computer-assisted surgery and kinematics (CAS), electromagnetic tracking (ETD), stereoradiography (SR), magnetic resonance imaging (MRI), kinematics from load applicator (LA), optical system (OPT), clinical device (CD) or miscellaneous (MISC). The reported knee kinematic: Anterior (ANT), Posterior (POST), Internal (INT), External (EXT), Valgus (VALG) or Varus (VAR). Y^p^ and Y^s^ distinguish between measured primary and secondary laxity, respectively while the Y^m^ distinguishes the use of a multiple load scenario.

### 3.1 Primary laxity under single load protocols–knee flexion specific

**FIGURE 2:**
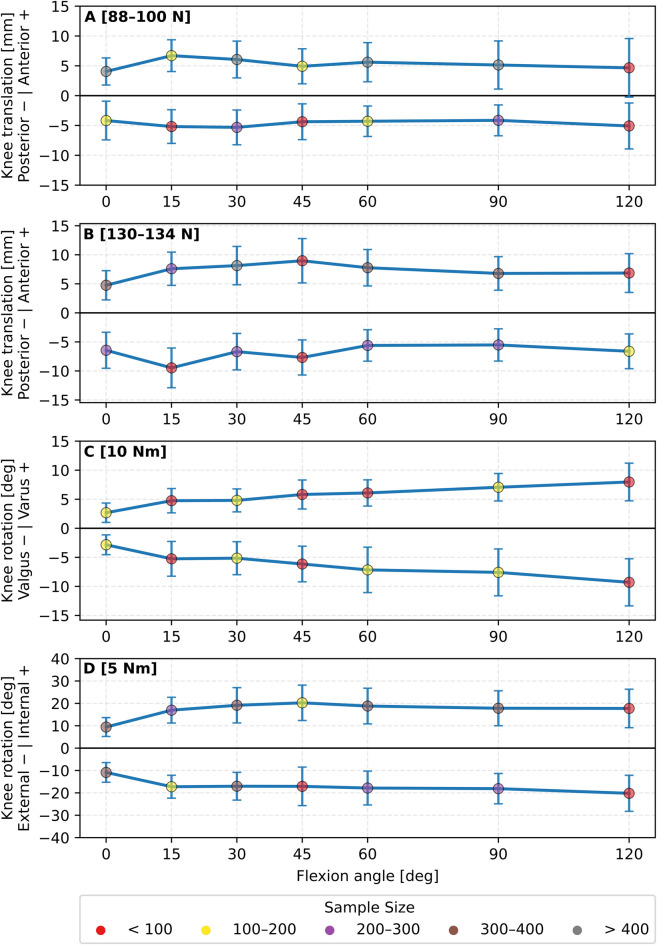
Overview of mean primary knee laxity of **(A)** anterior-posterior translation [88–100 N], **(B)** anterior-posterior translation [130–134 N], **(C)** valgus-varus rotation [10 Nm] and **(D)** internal-external rotation [5 Nm], with error bars indicating standard deviation. Data are grouped based on the identified load and knee flexion angles. Each data point is represented with a colour to illustrate the number of specimens embedded.

Of the included studies, 142 investigated primary laxity using at least one of the predefined knee flexion angles. For primary laxity, the load varied from 20 to 250 N for the anterior (98 studies), 20–200 N for the posterior directions (48 studies), 2 to 29 Nm for varus rotation (25 studies), 4 to 15 Nm for valgus rotation (31 studies), 1.5 to 10 Nm for internal rotation (55 studies) and 1.5 to 10 Nm for external rotation (46 studies). An overview of loads used to measure primary laxity are summarised (Supplementary Material S3). For those that satisfied the load-grouping criteria, it was condensed into each direction: anterior translation [88–100 N, 48 studies, 559 knees] and [130–134 N, 40 studies, 446 knees]; posterior translation [88–100 N, 22 studies, 218 knees] and [130–134 N, 22 studies, 267 knees]. Varus rotation [10 Nm, 13 studies, 175 knees] and valgus rotation [10 Nm, 15 studies, 196 knees]. Internal rotation [5 Nm, 39 studies, 463 knees] and external rotation [5 Nm, 31 studies, 387 knees]. Grouped translational and rotational primary knee laxity is summarised in Figure 2.

Compared with the 88–100 N loading condition, the 130–134 N condition resulted in greater anterior and posterior tibial translation across all knee flexion angles (Figures 2A,B). Based on raw data, anterior translation was 0.7–4.05 mm higher, with a mean difference of 1.96 mm, while posterior translation was 1.32–4.29 mm higher, with a mean difference of 2.24 mm. No formal hypothesis testing was performed. Varus and valgus rotation showed a clear trend toward increased laxity as a function of knee flexion (Figure 2C). At full knee extension, the rotational mean laxity was 2.7° and 2.8°, this changed to 7.9° and 9.2° at 120-degree knee flexion for varus and valgus, respectively. The internal and external rotation measured 9.4° and 10.8° at full extension and plateaued between 17° and 20° as knee flexion increased (Figure 2D).

**FIGURE 3:**
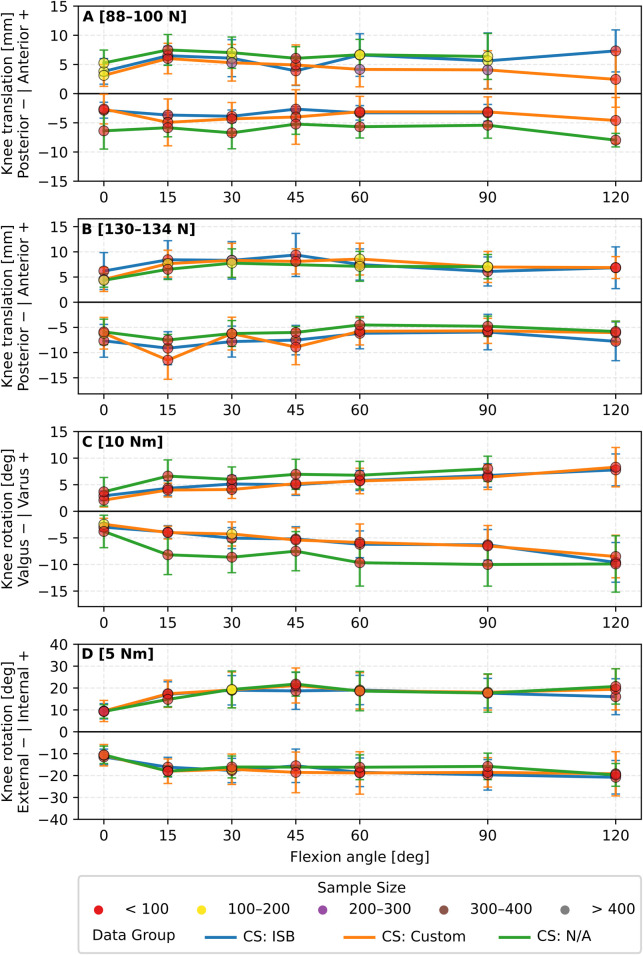
Overview of mean primary knee laxity of **(A)** anterior-posterior translation [88–100 N], **(B)** anterior-posterior translation [130–134 N], **(C)** valgus-varus rotation [10 Nm] and **(D)** internal-external rotation [5 Nm] using three different coordinate systems. Error bars indicate standard deviation. These coordinate systems are the International Society of Biomechanics (ISB, blue), custom defined (Custom, orange) and not available (N/A, green). Data are grouped based on the identified load and knee flexion angles. Each data point is represented with a colour to illustrate the number of specimens embedded.

**FIGURE 4:**
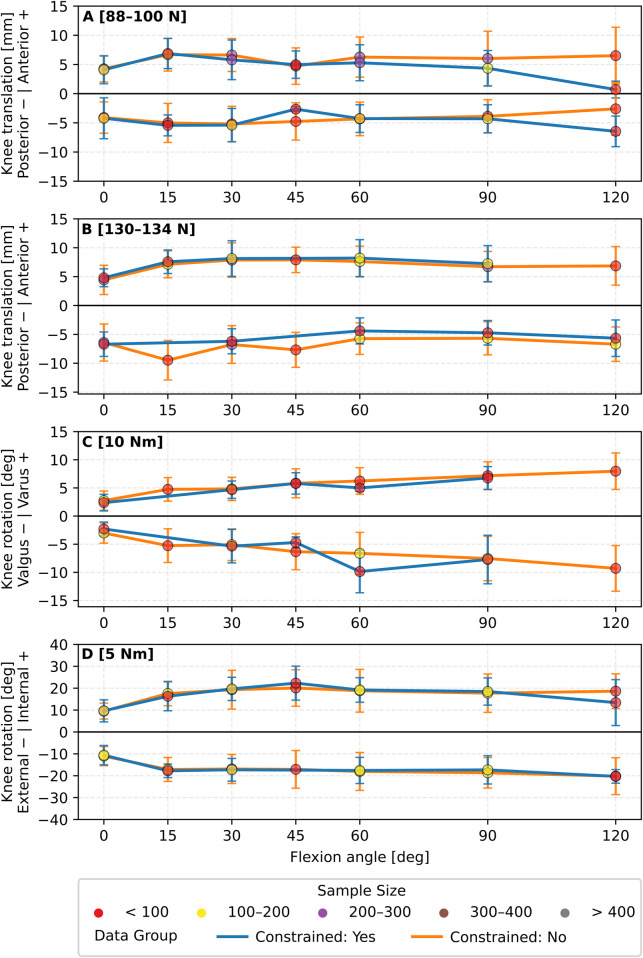
Overview of mean primary knee laxity of **(A)** anterior-posterior translation [88–100 N], **(B)** anterior-posterior translation [130–134 N], **(C)** valgus-varus rotation [10 Nm] and **(D)** internal-external rotation [5 Nm] using either constrained (blue) or unconstrained (orange) conditions. Error bars indicate standard deviation. Data are grouped based on the identified load and knee flexion angle.

Grouping primary laxity based on coordinate system definition, kinematic constraint conditions, and applied loads all influenced the overall laxity envelope (cf. Figures 3, 4). The data trend remained somewhat similar; however, the magnitude of the reported translations and rotations showed notable variability, especially with variation in coordinate system definition. This variability became particularly noticeable at mid-to-high knee flexion angles. For example, for the group of the [88–100N] anterior force, the reported anterior translation was 7.3 ± 3.7 mm at 120° knee flexion with the ISB recommendations (Figure 3). This value was 2.7 mm higher than the absolute value at the same angle once data were grouped independently of coordinate systems (Figure 2A). For the same load ([88–100N]) and knee flexion (120°), anterior translation measured 6.5 mm under constrained conditions and 0.7 mm when unconstrained (Figure 4). Oppositely, posterior translation at the same flexion angle was 2.6 mm constrained, 6.4 mm unconstrained. A full overview of absolute values and inter-condition differences is provided in Supplementary Material S4.

### 3.2 Specimen preparation, intactness and study characteristics

A total of 1,741 knees were prepared and analysed in the 161 included studies (Table 2). Specimen preparation characterisation showed a predominance of intact ligaments and partially intact muscles and skin (54%), while various other preparation approaches were used in the remaining specimens. This shows a high degree of diversity in how the anatomical integrity of the specimens was preserved in the experiments. In more than half of the studies, specimen sex was unknown, and only 6% reported information about BMI. All studies reported the inclusion of healthy intact knee specimens. With the fundamentals of these specimens as a basis for reporting laxity profiles, we found that the methodological effort made to determine that the specimens were, in fact, healthy, and the validity of these methods was usually low. The methodological description of how the knees were checked to rule out deformities (e.g., osteocytes, cartilage degeneration, meniscal lesions, and ligament tearing or fraying) was sparse. Overall, 94%, 93% and 80% of studies received low to medium or poorer ratings for methodological description, reproducibility and validation, respectively. A detailed overview of the methodological description and corresponding scores for each paper is provided in Supplementary Material (S5).

**TABLE 2:** Summary of experimental specimen conditions (N = 1741) reported in the included studies (n = 161).

Experimental specimen condition	No. of studies that adequately reported the condition	No. of specimens in the adequately reported studies
Mean age	112 (69%)	1,227 (70%)
Age range	127 (79%)	1,413 (81%)
Sex	71 (44%)	763 (49%)
BMI	9 (6%)	67 (4%)
Intactness	​	​
L	18 (12%)	198 (11%)
L/I	4 (2%)	58 (3%)
L/I/S	36 (22%)	395 (23%)
L/P	19 (12%)	187 (11%)
L/P/S	84 (52%)	903 (52%)

Specimen preparation was categorised based on the presence and condition of ligaments, muscles, skin and muscle tension. Ligaments only (L), Ligaments with Intact muscle (L/I), Ligaments with Intact muscle and Skin (L/I/S), Ligaments with Partial muscles (L/P) and Ligaments with Partial muscle and Skin (L/P/S).

### 3.3 Experimental testing and measuring characteristics

Knee laxity profiles were captured using various experimental methods (Table 3). Robotic arm systems emerged as the most frequently used approach for load applications, while other methods, such as universal testing machines and miscellaneous devices, were reported. Likewise, kinematic capturing systems predominantly relied on load applicator–based setups, although optical systems, electromagnetic tracking devices, and other modalities were observed. Variability was evident in the reporting of testing constraints, with studies being nearly evenly divided on whether constrained DOF were imposed or not (88 and 73 studies) during laxity measurements. The coordinate system recommended by the ISB was reported in 40 studies (24.8%) as the least used, while a custom-made coordinate system was reported in 57 studies (35.4%). The remaining 64 studies (39.8%) did not specify how the coordinate system was defined.

**TABLE 3:** Summary of experimental testing conditions reported in the included articles (n = 161).

​	Class	No. of studies out of total 161 included	Constrained		Coordinate system definition		
			Yes	No	ISB	Custom	N/A
Load Application	RA	105 (65.2%)	62 (59%)	43 (41%)	29 (27.6%)	43 (41%)	33 (31.4%)
	MISC	39 (24.2%)	14 (35.9%)	25 (64.1%)	8 (%)	12 (30.7%)	19 (48.7%)
	TM	15 (9.3%)	11 (73.3%)	4 (26.7%)	3 (20%)	2 (13.3%)	10 (66.7%)
	CD	3 (1.8%)	1 (33.3%)	2 (66.7%)	0	0	3 (100%)
Capturing system	LA	107 (66.4%)	65 (60.7%)	42 (39.3%)	29 (27.1%)	38 (35.5%)	40 (37.4%)
	OPT	22 (13.7%)	7 (31.8%)	15 (68.2%)	6 (27.3%)	10 (45.4%)	6 (27.3%)
	MISC	17 (10.5%)	9 (52.9%)	8 (47.1%)	2 (11.8%)	7 (41.2%)	8 (47%)
	ETD	6 (3.7%)	4 (66.7%)	2 (33.3%)	0	1 (16.7%)	5 (83.3%)
	SR	5 (3.1%)	1 (20%)	4 (80%)	3 (60%)	1 (20%)	1 (20%)
	CD	3 (1.8%)	1 (33.3%)	2 (66.7%)	0	0	3 (100%)
	CAS	2 (1.2%)	1 (50%)	1 (50%)	0	0	2 (100%)
	MRI	1 (0.6%)	1 (100%)	0	0	0	1 (100%)

Following abbreviations are used: Robot Arm (RA), Miscellaneous (MISC), Testing Machine (TM), Clinical Device (CD), From Load Applicator (LA), Optical System (OPT), Electromagnetic Tracking Device (ETD), Stereo Radiography (SR), Computer-assisted Surgery and Kinematics (CAS), Magnetic Resonance Imaging (MRI), Not Available (N/A), Custom defined coordinate system (Custom), Coordinate System of the International Society of Biomechanics (ISB) proposed by Grood and Suntay (1983), Constrained (Yes) and unconstrained (No) degrees of freedom during testing. Note that the percentages do not match fully as a few studies used multiple load application and capturing system classes; Jette et al. (2019) used MISC and TM in the load application; Küpper et al. (2022) used ETD and MRI in the capturing system and Senioris et al. (2017) used CD and SR in the capturing system.

## 4 Discussion

This review provides a comprehensive overview of *in vitro* measurements of intact knee laxity and highlights the considerable variability observed across studies, even when comparable experimental setups were used. The finding that similar testing conditions can produce significantly different laxity values supports the need for greater methodological consistency and transparent reporting. Anterior-posterior was, by far, the most measured direction, followed by internal-external and varus-valgus laxity. Here, the commonly used experimental setup was to apply loads with a robot arm while measuring kinematics from the load application itself. Despite the experiment setup being identical in many cases, significant discrepancies were noted in the applied loads, specimen preparation, knee intactness, coordinate system definition, and kinematically constrained boundary conditions applied to the specimens (cf. data in Tables 1–3). These inconsistencies were concomitant with differences in laxity profiles (cf. Figures 2–4). Hence, Figure 2 represents a pooled overview of *in vitro* knee laxity data collected under varying methodological conditions rather than a definitive reference curve for a healthy intact knee’s laxity profile. This methodological heterogeneity is a key finding, and aligned well with previous laxity reviews (Ferle et al., 2019). This clearly indicates a need for standardised guidelines to ensure reliable, credible and reproducible results in future research to improve scientific impact.

### 4.1 Coordinate system definition

A considerable portion of the observed variability may stem from how joint motion is expressed. Differences in the defined coordinate system can modify both the laxity measured and, in some cases, the direction of measured laxity, particularly across different knee flexion angles (cf. Figure 3). This is because different coordinate systems can vary in how anatomical axes are defined and oriented. Thus, small differences in axis orientation or origin placement can result in markedly different interpretations of the same physical motion (Beardsley et al., 2007; Roos et al., 2005). For example, an anterior translation in one coordinate system might partially project into a medial-lateral direction. This sensitivity becomes increasingly relevant as joint orientation changes with flexion. Notably, the distribution of data across coordinate systems is not uniform, and the apparent variability may also reflect differences in data availability rather than actual biomechanical differences in the measured laxity profile (cf. Figure 3). Surprisingly, most studies failed to report how their coordinate system was constructed (cf. Table 3), and the Grood and Suntay (Grood and Suntay, 1983) standard recommended by the ISB since 1983 was the least used. Obviously, papers published before ISB recommendations cannot comply with these standards. Nevertheless, this is concerning because well-defined coordinate systems are essential for reproducibility and for enabling meaningful comparison of laxity response across studies. Within-subjects study designs do have advantages, such as comparing intact knees with sequential cutting; however, inconsistent experimental boundary conditions make it questionable to compare the effects of different surgical procedures reported between studies. As recently discussed, simply relabelling x, y, z, roll, pitch, yaw conventions are not equivalent to using proper biomechanical coordinate systems (Colbrunn et al., 2024). We, therefore, propose that explicit reporting and adherence to ISB recommendations should become standard practice to improve the comparability of laxity measurements.

### 4.2 Kinematic constraints

Kinematic constraints determine how the applied load is shared between primary and secondary DOFs, and the selected testing strategy has a major influence on the resulting laxity profiles. To illustrate this dependency, laxity profiles were categorised into constrained and unconstrained testing strategies (cf. Figure 4). For anterior-posterior translation at [88–100 N], differences between constrained and unconstrained testing were minimal at flexion angles up to 90° (≤1.7 mm for anterior and ≤2.1 mm for posterior) but increased at 120-degree flexion (5.8 mm and 3.9 mm, respectively), see Supplementary Material S4. This pattern likely reflects the dominant role of the ACL and posterior cruciate ligament (PCL) in restraining anterior-posterior motion at lower flexion angles, where ligament tension limits secondary motion. At higher flexion, reduced ACL and PCL tension diminishes their restraining capacity, while other structures (e.g., collateral ligaments) become taut, allowing more secondary laxity. Comparable effects are evident in the dataset, for example, internal rotation at 120-degree flexion (5.2°), and valgus rotation at 60-degree flexion (3.2°). This directional dependency emphasises the importance of careful consideration of constraint effects in relation to both load direction and joint biomechanics. Despite the presence of secondary motion, the variability observed was not as substantial as expected. The only setting that indicates a noteworthy difference was the laxity seen for the anterior translation in the 88–100 N loading group at 120° knee flexion which had a difference of 5.8 mm (cf. Figure 4). This may suggest that constraining DOFs is not inherently detrimental to the measurement of joint laxity; however, very little is known in this area. In fact, *in situ* loads of the ACL have been observed to increase in contribution when the kinematics are constrained to one DOF compared to 5 DOFs (Livesay et al., 1997; Takai et al., 1993). This indicates that kinematically constraining the DOFs is more effective in engaging the ligament, thereby allowing for a more isolated assessment of the mechanical response. Oppositely, a constrained setup might not correctly replicate real-world scenarios when a clinical laxity test is performed *in vivo*, and the correct choice may be dependent on the research question.

### 4.3 Demographic reporting

Limited and inconsistent reporting of specimen age, sex, and BMI restricts stratified analyses and hinders aggregation and comparison of findings across studies. This shortcoming was common across the included literature, with key demographic information frequently missing (Table 2, Supplementary Material S2). Minimal studies exclusively examined one specific sex, with males (11 out of 161) (Boguszewski et al., 2015; Branch et al., 2017; Henke et al., 2024; Kim et al., 2015; Li et al., 1999; Markolf et al., 2008; Nitri et al., 2016; Rasmussen et al., 2016; Sasaki et al., 2007; Serbino et al., 2015; Wilhelm et al., 2023) being more common than females (2 out of 161) (Boguszewski et al., 2015; Küpper et al., 2022). The remaining studies used a mixed sex cohort and lacked stratification. Furthermore, the age distribution was heavily skewed toward older donors, with one out of four studies reporting mean specimen age below 55 years, and BMI was only reported in nine studies (Andreassen et al., 2021; Beel et al., 2024; Branch et al., 2017; Kennedy et al., 2014a; Kennedy et al., 2014b; Pedersen et al., 2019; Reynolds et al., 2017; Roth et al., 2015; Wang et al., 2014) (see Supplementary Material S2 for full data overview). One study found that sex contributed significantly to joint laxity, with female knees being laxer (Boguszewski et al., 2015), but none of the studies explicitly investigated age contribution. Thus, very little is known about the biological relationship between demographic factors and knee laxity. Associations are well established in other conditions, such as OA, where prevalence varies with sex (Segal et al., 2024), age (Shane Anderson and Loeser, 2010) and BMI (Solanki et al., 2023). Unfortunately, despite the large volume of available data, the inconsistent reporting of demographics means that the dataset lacks the necessary granularity to disentangle and evaluate sex- and age-dependent trends in knee laxity. This is particularly problematic because intersubject variability stemming from factors such as age, sex, BMI, and injury history could significantly impact knee laxity and represents a critical dimension of joint biomechanics (Mouton et al., 2015; Cook et al., 2014).However, as demonstrated in this review, the current methodological rigor is fragmented and the possibility of isolating these biological factors are limited (Ferle et al., 2019). In essence, the methodological “noise” introduced by heterogeneity currently overwhelms the biological “signal” of true intersubject differences. To overcome these issues, standardised reporting should be prioritised in future studies (see recommendations).

### 4.4 Specimen screening and preparation

Another key finding of this review was the inconsistent specimen screening and preparation procedures. While most studies claimed to assess the integrity of joint structures such as cartilage, menisci and ligaments, they rarely specified exactly what the procedure included. For example, some studies stated that they used the medical record for screening (Wijdicks et al., 2013a; Wijdicks et al., 2013b) or checked that the knees were grossly intact (Bae et al., 2008; Boguszewski et al., 2015; Geeslin et al., 2018). Others evaluated the knees with imaging techniques (Gabriel et al., 2004; Harner et al., 2000; Kato et al., 2013; Lording et al., 2017), arthroscopy (Fayed et al., 2022; Liu et al., 2014; Liu et al., 2020; Nielsen et al., 2018) but still inconsistently providing the resulting OA grading of the cadaveric specimens (Branch et al., 2017; He et al., 2023). This lack of methodological coherence and transparency limits the ability to replicate the screening procedure in another population subset (see Supplementary Material S5 for full data overview). As a result, it is possible that pathological knees were inadvertently included. Moreover, variability in specimen preparation introduces a significant source of volatility in the reported laxity. The majority retained partially intact muscle and skin, but the preparation ranged from ligament only to those with intact or partially intact preserved soft tissue (Table 2). These differences are not trivial, as the presence or absence of periarticular tissues can meaningfully alter joint biomechanics by affecting passive tension, load distribution, and stabilising constraints; consequently, different laxities have been observed (Beidokhti et al., 2018). When these factors are inconsistently reported, isolating the actual laxity profile becomes difficult, as insufficient data is available for certain scenarios. Thus, without standardised protocols, comparisons across studies remain confounded, and any attempt to define normative laxity profiles risks being erroneous and skewed by methodological artefacts rather than true biological variation.

### 4.5 Limitations

Although this scoping review provides a comprehensive synthesis of knee joint laxity data, significant limitations remain that constrain the interpretability of the findings. The wide heterogeneity in experimental methodologies, inconsistent reporting of coordinate systems, kinematic constraints, unclear screening protocols and specimen preparation practices introduce substantial variability. This variability precludes the use of meta-analysis, as combining such disparate data would not yield valid quantitative conclusions, consistent with findings from previous reviews that also reported high heterogeneity in translational and rotational measurements of the knee joint laxity (Ferle et al., 2019). Demographic gaps further limit the ability to explore biologically relevant patterns. Consequently, the presented pooled laxity profiles likely reflect underlying biomechanical trends but also methodological artefacts and should be interpreted appropriately.

The lack of consensus outlined limits the ability to make comparisons between studies and to propose clinical guidelines. Yet, the documented laxity profiles (Figure 2) still provide information on how knee laxity varies with flexion angle. However, only data from healthy *in vitro* conditions were synthesised. Consequently, it remains uncertain whether the flexion angles that exhibit the greatest physiological laxity also represent the most diagnostically sensitive position for detecting ligament injuries. Instead, it is possible that the optimal clinical testing angle is the one where injury-induced changes in laxity are most pronounced, i.e., where the difference between intact and injured states is greatest, thereby enhancing detectability. The considerable methodological heterogeneity complicates cross-study comparisons. As a result, drawing definitive conclusions regarding clinical guidance would be unsound, as it would essentially involve the synthesis of non-comparable datasets. This highlights a key limitation not only in the present review but in knee laxity literature. Therefore, guidelines are needed to standardise reporting and to improve methodological transparency. Our guidelines, as derived from our analysis and consideration of both common clinically and experimentally relevant procedures, are provided below:

### 4.6 Guidelines for healthy *in vitro* knee laxity testing

Based on our scoping review and the challenges identified in current practices, we propose the following guidelines to improve the standardisation and reporting of *in vitro* knee laxity:

#### 4.6.1 Specimen reporting

- Report the age (mean and standard deviation) and age range.
- Provide a clear breakdown of sex (sex balance is encouraged).
- If available, provide information about BMI.

#### 4.6.2 Specimen knee intactness check

- Always provide a clear OA check. We recommend that researchers follow the current clinical guidelines for radiographic OA check and include a relevant OA score, e.g., Kellgren-Lawrence, Outerbridge and ensure that all included healthy knees are scored as healthy on the chosen scale.
- Check and describe the integrity of the ligaments and menisci. We recommend performing an arthroscopic procedure to visualise and investigate all relevant structures (Ward and Lubowitz, 2013).
- Check and describe the integrity of the bones. We recommend performing an X-ray to determine the formation of osteophytes, periarticular ossicles, cysts and subchondral sclerosis (Kellgren and Lawrence, 1957).
- State how the ligaments, muscles, and skin are prepared and preserved.
  - Ligaments only (L).
  - Ligaments and partial muscles (L/P).
  - Ligaments and intact muscle (L/I).
  - Ligaments and partial muscle and skin (L/P/S).
  - Ligaments and intact muscle and skin (L/I/S).
  - Ligaments and intact skin, muscle and applied tensions (L/S/T).
- If surgical procedures (e.g., osteotomy) are performed before testing the intact state, this should be described. This is not recommended, and we always encourage to test the intact state without interfering with the joint.

If specimen information is unknown or unavailable, this should be stated in the manuscript, as failing to clarify the conditions of the specimens will reduce transparency and credibility. Specimen knee intactness checking should always be performed subsequent to testing to not interfere with the integrity of the intact knee joint. Thus, exclusion of pathological knees is also done retrospectively. When a pathological knee is included, present the data in the Supplementary Material and exclude it from the overall analysis.

#### 4.6.3 Experimental testing and measuring conditions

The experimental testing and measuring guidelines are formulated based on our observations and previous best-practices summary for conducting *in vitro* knee biomechanical testing (Colbrunn et al., 2024). Our guidelines are like previous with the most important aspects highlighted here, see this best-practices overview for a more in-depth description (Colbrunn et al., 2024).

- State the equipment used in the experiment.
  - State how the load (e.g., dynamic, static, quasistatic etc.) was applied and the magnitude.
  - State the root mean square error between the desired and actual load.
  - State how joint kinematics were recorded (e.g., robotic arm, universal testing machine, optical motion capture, etc.).
- Description of the knee boundary conditions (i.e., constrained or unconstrained).
  - In a constrained setup, some or all other DOFs are restricted during testing either by hardware or software. Provide a description of which DOFs are constrained and which are not.
  - In an unconstrained setup, all secondary DOFs are left to move naturally in response to the mechanical load.
- Description of coordinate system definition.
  - We recommend using the ISB (Grood and Suntay, 1983) definitions as a joint coordinate system.

#### 4.6.4 Laxity reporting

- Always include the actual values (mean and standard deviation) of data points that produced graphical content. If not within the main manuscript, Supplementary Material is necessary. See our Supplementary Material S4 for an example; this Supplementary Material has all raw data to enable calculations of graphical content but also differences in laxity between each reported condition.
- If coupled laxity is reported e.g., anterior and posterior translation cannot be isolated, always include the results of the individual directions. We experienced quite a few articles reporting coupled laxity without providing the isolated results.
- If side-to-side differences are reported, always include the actual values (mean and standard deviation) of the intact condition to allow for re-calculation. Supplementary Material is recommended.
- If the purpose of the study is to investigate new surgical procedures, always include the laxity measurements of the intact knee. We experienced many articles to be excluded as no laxity measurement of the intact knee was reported.
- Where possible, stratify the analysis by sex and age.
  - If this is outside the scope, we recommend Supplementary Material with the stratified laxity data reported (mean and standard deviation).
  - Preferably, stratification can also be done on BMI.
- Indicate if the data provided is primary or secondary laxity.
  - Primary laxity refers to joint movement in the same direction as the applied load and is studied only under one specific load.
  - Secondary laxity refers to movement in different directions than the applied load or if multiple loads are used simultaneously.

By adhering to these guidelines, it will be possible to facilitate meta-analyses and improve the comparability of results across different studies. With experimental conditions kept constant comparison of surgical techniques can be established across studies, providing more sound evidence. Clear stratification of sex and age when reporting laxity enables the possibility of understanding how laxity may be age- and sex-dependent. By complying to these recommendations in the community clinical applications and overall scientific knowledge can be improved collectively.

## 5 Conclusions and future directions

This study systematically reviewed *in vitro* knee laxity testing literature from its beginning, dating back to the 1970s (Hsieh and Walker, 1976; Markolf et al., 1976) and until the current stage of knowledge. It was shown that most studies applied loads with a robot arm while measuring kinematics from the load application mechanism. Further exploration led to findings of high heterogeneity across studies in terms of applied loads, specimen preparation and reporting, specimen intactness, kinematic constraints and coordinate system definition. Consequently, the established primary laxity profiles of the intact knees should be scrutinised. Thus, careful consideration of testing protocols and methods for specimen preparation and preservation must be taken, and this work serves as a guide for those undertaking such studies in the future. Standardisation of knee laxity testing and reporting will enable easier comparison across studies and will undoubtedly accelerate advancements in surgical techniques and overall understanding of human knee laxity.

Looking ahead, emerging *in vivo* techniques (Küpper et al., 2007; Kümmerlin et al., 2022; Küpper et al., 2016) may enable nonterminal, longitudinal assessment of knee laxity. This may include tracking joint behaviour over time to evaluate recovery, surgical outcomes, or the progression of laxity. While such technologies hold great potential, they must be anchored in robust, validated reference data. As technologies mature, the benchmarks synthesised in this review can serve as a critical foundation for interpreting future findings, guiding surgical decision-making and standardising clinical evaluations.
