# Supplementary material for: A scoping review and guide for in vitro healthy human knee joint laxity
Source: Front Bioeng Biotechnol. 2026 Mar 18;14:1741003. doi: 10.3389/fbioe.2026.1741003 (PMC13038627; doi:10.3389/fbioe.2026.1741003)
Supplement: Supplementary file 2 [file Table1.docx]

Supplementary Material

Bo Eitel Seiferheld^1,2,3^, Martin Lindvad Pedersen^1,3^, Ilias Theodorakos^1^, Brett Michael Musolf^1,2^, Morten Bilde Simonsen^1,2^, Michael Skipper Andersen^1,2^, Mohammadjavad (Matin) Einafshar^1,2*^

^1^Department of Materials and Production, Aalborg University, Fibigerstræde 16, 9220 Aalborg East, Denmark.

^2^Center for Mathematical Modeling of Knee Osteoarthritis, Department of Materials and Production, Aalborg University, Fibigerstræde 16, 9220 Aalborg East, Denmark

^3^Shared first authorship.

# Search Query 30-10-2024

This supplementary document provides an overview of the systematic literature search strategy, detailing the different aspects of the review and their respective search terms. Within each aspect, terms were combined using the Boolean operator OR to capture all relevant variations, with the total hits presented for each aspect. The aspects were then combined using the Boolean operator AND to iteratively refine the search and identify studies that met all cumulative criteria. The search was conducted across three digital databases: PubMed, Embase, and Web of Science, spanning all publications up to 30 October 2024.

## PubMed.

| NO. | Aspect/level | Search terms | Hits |
| --- | --- | --- | --- |
| 1 | Biomechanical Phenomena | "Biomechanical Phenomena"[Mesh] OR  "Mechanical Phenomena"[Mesh] OR  "Human biomechanics"[Title/Abstract] | 970.445 |
| 2 | Knee Joint and Participant | "Knee/surgery"[Mesh] OR  "Knee Joint/surgery"[Mesh] OR  "Knee Joint/diagnostic imaging"[Mesh] OR  "Knee Injuries/diagnostic imaging"[Mesh] OR  "Knee Injuries/surgery"[Mesh] OR  "Osteoarthritis, Knee"[Mesh] OR  "Knee Joint/physiology"[Mesh] OR  "Knee Joint"[Title/Abstract] OR  "Normal knee"[Title/Abstract] OR  "Healthy knee"[Title/Abstract] OR  "Human knee"[Title/Abstract] | 101.836  18.411 |
| 3 | Measurement | "Measurement"[Title/Abstract] OR  "Measure"[Title/Abstract] OR  "Range of Motion, Articular"[Mesh] OR  "kinematics"[Title/Abstract] OR  "Varus"[Title/Abstract] OR  "Valgus"[Title/Abstract] OR  "Predictive Value of Tests" [Mesh] OR  "Internal rotation"[Title/Abstract] OR  "External rotation"[Title/Abstract] OR  "Rotational"[Title/Abstract] OR  "Displacement"[Title/Abstract] OR  "Anteroposterior"[Title/Abstract] OR  "Anterior"[Title/Abstract] OR  "Posterior"[Title/Abstract] OR  "Tilting"[Title/Abstract] OR  "Medio-lateral"[Title/Abstract] | 2.366.172  12.551 |
| 4 | Validation and methods | "device"[Title/Abstract] OR  "Validation"[Title/Abstract] OR  "In-vitro validation"[Title/Abstract] OR  "Robotics*" [Mesh] OR  "Equipment" [Title/Abstract] OR  "Machine" [Title/Abstract] OR  "Tool" [Title/Abstract] OR  "Apparatus" [Title/Abstract] OR  "Proof of concept" [Title/Abstract] OR  "Verification"[Title/Abstract] OR  "Equipment Design" [Mesh] | 2.043.132  2.571 |
| 5 | Laxity | "Instability"[Title/Abstract] OR  "Laxity"[Title/Abstract] OR  "Joint Instability/diagnosis*"[Mesh] OR  "Balancing"[Title/Abstract] OR  "Imbalance"[Title/Abstract] OR  "Stability"[Title/Abstract] OR  "Joint Instability/physiopathology" [Mesh] OR  "Stiffness"[Title/Abstract] OR  "Joint Instability/etiology*" [Mesh] OR  "Malalignment” [Title/Abstract] OR  "Malrotation" [Title/Abstract] OR  "Joint Rigidity" | 980,849  842 |

### PubMed Query:

((((("Biomechanical Phenomena"[MeSH Terms] OR "Mechanical Phenomena"[MeSH Terms] OR "Human biomechanics"[Title/Abstract]) AND (1000/1/1:2024/10/30[pdat])) AND ("Knee/surgery"[Mesh] OR "Knee Joint/surgery"[Mesh] OR "Knee Joint/diagnostic imaging"[Mesh] OR "Knee Injuries/diagnostic imaging"[Mesh] OR "Knee Injuries/surgery"[Mesh] OR "Osteoarthritis, Knee"[Mesh] OR "Knee Joint/physiology"[Mesh] OR "Knee Joint"[Title/Abstract] OR "Normal knee"[Title/Abstract] OR "Healthy knee"[Title/Abstract] OR "Human knee"[Title/Abstract] AND (1000/1/1:2024/10/30[pdat]))) AND ("Measurement"[Title/Abstract] OR "Measure"[Title/Abstract] OR "Range of Motion, Articular"[Mesh] OR "kinematics"[Title/Abstract] OR "Varus"[Title/Abstract] OR "Valgus"[Title/Abstract] OR "Predictive Value of Tests" [Mesh] OR "Internal rotation"[Title/Abstract] OR "External rotation"[Title/Abstract] OR "Rotational"[Title/Abstract] OR "Displacement"[Title/Abstract] OR "Anteroposterior"[Title/Abstract] OR "Anterior"[Title/Abstract] OR "Posterior"[Title/Abstract] OR "Tilting"[Title/Abstract] OR "Medio-lateral"[Title/Abstract] AND (1000/1/1:2024/10/30[pdat]))) AND ("device"[Title/Abstract] OR "Validation"[Title/Abstract] OR "In-vitro validation"[Title/Abstract] OR "Robotics*" [Mesh] OR "Equipment" [Title/Abstract] OR "Machine" [Title/Abstract] OR "Tool" [Title/Abstract] OR "Apparatus" [Title/Abstract] OR "Proof of concept" [Title/Abstract] OR "Verification"[Title/Abstract] OR "Equipment Design" [Mesh] AND (1000/1/1:2024/10/30[pdat]))) AND ("Instability"[Title/Abstract] OR "Laxity"[Title/Abstract] OR "Joint Instability/diagnosis*"[Mesh] OR "Balancing"[Title/Abstract] OR "Imbalance"[Title/Abstract] OR "Stability"[Title/Abstract] OR "Joint Instability/physiopathology" [Mesh] OR "Stiffness"[Title/Abstract] OR "Joint Instability/etiology*" [Mesh] OR "Malalignment" [Title/Abstract] OR "Malrotation" [Title/Abstract] OR "Joint Rigidity" AND (1000/1/1:2024/10/30[pdat])).

## Embase.

| NO. | Aspect/level | Search terms | Hits |
| --- | --- | --- | --- |
| 0 | Biomechanical Phenomena | "Biomechanics"/de OR  "Mechanical Phenomena" OR  "biomechanics" | 202.836 |
| Joint instability  1 | Knee Joint and Participant | 'knee'/de OR  'knee joint':ti OR  'knee joint':ab OR  'knee joint':kw OR  'osteoarthritis':ti OR  'osteoarthritis':ab OR  'osteoarthritis':kw OR  'normal knee':ti OR  'healthy knee':ti OR  'normal knee':ab OR  'healthy knee':ab OR  'normal knee':kw OR  'healthy knee':kw | 220.796  18.060 |
| 2 | Measurement | 'measurement'/de OR  'range of motion'/de OR  'kinematics'/de OR  'varus':ti OR  'varus':ab OR  'varus':kw OR  'valgus':ti OR  'valgus':ab OR  'valgus':kw OR  'predictive value of tests':ti OR  'predictive value of tests':ab OR  'predictive value of tests':kw OR  'internal rotation':ti OR  'internal rotation':ab OR  'internal rotation':kw OR  'external rotation':ti OR  'external rotation':ab OR  'external rotation':kw OR  'displacement':ti OR  'displacement':ab OR  'displacement':kw OR  'anterior-posterior':ti OR  'anterior-posterior':ab OR  'anterior-posterior':kw OR  'tilting':ti OR  'tilting':ab OR  'tilting':kw OR  'medio-lateral':ti OR  'medio-lateral':ab OR  'medio-lateral':kw | 395.015323  6.466 |
| 3 | Validation and methods | 'device'/dv OR  'equipment design'/de OR  'equipment':ti OR  'equipment':ab OR  'equipment':kw OR  'machine*':ti OR  'machine*':ab OR  'machine*':kw OR  'tool*':ti OR  'tool*':ab OR  'tool*':kw OR  'apparatus':ti OR  'apparatus':ab OR  'apparatus':kw OR  'validation':ti OR  'validation':ab OR  'validation':kw OR  'proof of concept':ti OR  'proof of concept':ab OR  'proof of concept':kw OR  'verification':ti OR  'verification':ab OR  'verification':kw OR  'robotic*':ti OR  'robotic*':ab OR  'robotic*':kw | 2.657.954  887 |
| 4 | Laxity | 'instability':ti OR  'instability':ab OR  'instability':kw OR  'malalignment':ti OR  'malalignment':ab OR  'malalignment':kw OR  'malrotation':ti OR  'malrotation':ab OR  'malrotation':kw OR  'laxity':ti OR  'laxity':ab OR  'laxity':kw OR  'balancing':ti OR  'balancing':ab OR  'balancing':kw OR  'imbalance':ti OR  'imbalance':ab OR  'imbalance':kw OR  'stability':ti OR  'stability':ab OR  'stability':kw OR  'stiffness':ti OR  'stiffness':ab OR  'stiffness':kw OR  'joint instability':ti OR  'joint instability':ab OR  'joint instability':kw OR  'joint rigidity':ti OR  'joint rigidity':ab OR  'joint rigidity':kw | 1.162.129  304 |

### Embase Query:

('biomechanics'/de OR 'mechanical phenomena' OR 'biomechanics') AND ('knee'/de OR 'knee joint':ti OR 'knee joint':ab OR 'knee joint':kw OR 'osteoarthritis':ti OR 'osteoarthritis':ab OR 'osteoarthritis':kw OR 'normal knee':ti OR 'healthy knee':ti OR 'normal knee':ab OR 'healthy knee':ab OR 'normal knee':kw OR 'healthy knee':kw) AND ('measurement'/de OR 'range of motion'/de OR 'kinematics'/de OR 'varus':ti OR 'varus':ab OR 'varus':kw OR 'valgus':ti OR 'valgus':ab OR 'valgus':kw OR 'predictive value of tests':ti OR 'predictive value of tests':ab OR 'predictive value of tests':kw OR 'internal rotation':ti OR 'internal rotation':ab OR 'internal rotation':kw OR 'external rotation':ti OR 'external rotation':ab OR 'external rotation':kw OR 'displacement':ti OR 'displacement':ab OR 'displacement':kw OR 'anterior-posterior':ti OR 'anterior-posterior':ab OR 'anterior-posterior':kw OR 'tilting':ti OR 'tilting':ab OR 'tilting':kw OR 'medio-lateral':ti OR 'medio-lateral':ab OR 'medio-lateral':kw) AND ('device'/dv OR 'equipment design'/de OR 'equipment':ti OR 'equipment':ab OR 'equipment':kw OR 'machine*':ti OR 'machine*':ab OR 'machine*':kw OR 'tool*':ti OR 'tool*':ab OR 'tool*':kw OR 'apparatus':ti OR 'apparatus':ab OR 'apparatus':kw OR 'validation':ti OR 'validation':ab OR 'validation':kw OR 'proof of concept':ti OR 'proof of concept':ab OR 'proof of concept':kw OR 'verification':ti OR 'verification':ab OR 'verification':kw OR 'robotic*':ti OR 'robotic*':ab OR 'robotic*':kw) AND ('instability':ti OR 'instability':ab OR 'instability':kw OR 'malalignment':ti OR 'malalignment':ab OR 'malalignment':kw OR 'malrotation':ti OR 'malrotation':ab OR 'malrotation':kw OR 'laxity':ti OR 'laxity':ab OR 'laxity':kw OR 'balancing':ti OR 'balancing':ab OR 'balancing':kw OR 'imbalance':ti OR 'imbalance':ab OR 'imbalance':kw OR 'stability':ti OR 'stability':ab OR 'stability':kw OR 'stiffness':ti OR 'stiffness':ab OR 'stiffness':kw OR 'joint instability':ti OR 'joint instability':ab OR 'joint instability':kw OR 'joint rigidity':ti OR 'joint rigidity':ab OR 'joint rigidity':kw) AND [01-01-1000]/sd NOT [30-10-2024]/sd.

## Web of Science.

| NO. | Aspect/level | Search terms | Hits |
| --- | --- | --- | --- |
| 0 | Biomechanical Phenomena | TS= ("Biomechanical Phenomena") OR  TS= ("mechanical Phenomena") OR  TS=("biomechanics") | 59.004 |
| 1 | Knee Joint and Participant | TS= ("Knee/surgery") OR  TS= ("Knee Joint/surgery") OR  TS= ("Knee Joint/diagnostic imaging") OR  TS= ("Osteoarthritis, Knee") OR  TS= ("Knee Joint") OR  TS= ("Human knee") OR  TS= ("Normal knee") OR  TS= ("Healthy knee") | 34.351  2,900 |
| 2 | Measurement | TS= ("Measurement") OR  TS= ("Range of Motion, Articular") OR  TS= ("Kinematics") OR  TS= ("Varus") OR  TS= ("Valgus") OR  TS= ("Internal rotation") OR  TS= ("External rotation") OR  TS= ("Displacement") OR  TS= ("Tilting") OR  TS= ("Anterior posterior") OR  TS= ("Medio-lateral") | 1.931.060  1,627 |
| 3 | Validation and methods | TS= ("apparatus") OR  TS= ("non-invasive validation") OR  TS= ("Robotics ") OR  TS= ("machine") OR  TS= ("equipment") OR  TS= ("device") OR  TS= ("Validation") OR  TS= ("Verification") OR  TS= ("Proof of concept") | 2.920.968  227 |
| 4 | Laxity | TS= ("Laxity") OR  TS= ("Instability") OR  TS= ("Joint Instability") OR  TS= ("Balancing") OR  TS= ("Imbalance") OR  TS= ("Stability") OR  TS= ("Malalignment") OR  TS= ("Joint Instability") OR  TS= ("Joint Rigidity") OR  TS= ("Joint Instability") OR  TS= ("Stiffness") OR  TS= ("Malrotation") | 2.927.048  76 |

### Web of Science Query:

((TS= ("Biomechanical Phenomena") OR TS= ("mechanical Phenomena") OR TS=("biomechanics")) AND (TS= ("Knee/surgery") OR TS= ("Knee Joint/surgery") OR TS= ("Knee Joint/diagnostic imaging") OR TS= ("Osteoarthritis, Knee") OR TS= ("Knee Joint") OR TS= ("Human knee") OR TS= ("Normal knee") OR TS= ("Healthy knee")) AND (TS= ("Measurement") OR TS= ("Range of Motion, Articular") OR TS= ("Kinematics") OR TS= ("Varus") OR TS= ("Valgus") OR TS= ("Internal rotation") OR TS= ("External rotation") OR TS= ("Displacement") OR TS= ("Tilting") OR TS= ("Anterior posterior") OR TS= ("Medio-lateral")) AND (TS= ("apparatus") OR TS= ("non-invasive validation") OR TS= ("Robotics ") OR TS= ("machine") OR TS= ("equipment") OR TS= ("device") OR TS= ("Validation") OR TS= ("Verification") OR TS= ("Proof of concept")) AND (TS= ("Laxity") OR TS= ("Instability") OR TS= ("Joint Instability") OR TS= ("Balancing") OR TS= ("Imbalance") OR TS= ("Stability") OR TS= ("Malalignment") OR TS= ("Joint Instability") OR TS= ("Joint Rigidity") OR TS= ("Joint Instability") OR TS= ("Stiffness") OR TS= ("Malrotation"))) AND DOP=(1000-01-01/2024-10-30).
