# Supplementary material for: A scoping review and guide for in vitro healthy human knee joint laxity
Source: Front Bioeng Biotechnol. 2026 Mar 18;14:1741003. doi: 10.3389/fbioe.2026.1741003 (PMC13038627; doi:10.3389/fbioe.2026.1741003)
Supplement: Supplementary file 4 [file Table3.docx]

Supplementary Material

Bo Eitel Seiferheld^1,2,3^, Martin Lindvad Pedersen^1,3^, Ilias Theodorakos^1^, Brett Michael Musolf^1,2^, Morten Bilde Simonsen^1,2^, Michael Skipper Andersen^1,2^, Mohammadjavad (Matin) Einafshar^1,2*^

^1^Department of Materials and Production, Aalborg University, Fibigerstræde 16, 9220 Aalborg East, Denmark.

^2^Center for Mathematical Modeling of Knee Osteoarthritis, Department of Materials and Production, Aalborg University, Fibigerstræde 16, 9220 Aalborg East, Denmark

^3^Shared first authorship.

# Grouping of load and knee flexion angles.

This supplementary material provides an overview of the grouping of loads and knee flexion angles as performed in the systematic review. Grouping was based on data available to only provide knee laxity information from the generally applied loads and measured flexion angles from literature. Force data were grouped into knee samples tested at 88-100 N and 130-134 N, while moment data were grouped into 5 Nm and 10 Nm, as these ranges contained most of the reported values (**Supplementary Figure 1.1 – 1.6**). An equivalent procedure was applied for specific knee flexion angles, where 0°, 15°, 30°, 45°, 60°, 90°, and 120° were used (**Supplementary Figure 1.7**). Note that the bar annotations report the number of distinct studies at each specific load or angle. Because some studies report results at multiple loads or angles, the total sum of the annotation values across bars can exceed the total number of unique studies within a panel. Accordingly, annotations should be interpreted as per-category study counts, not as totals of unique studies/authors for the panel.


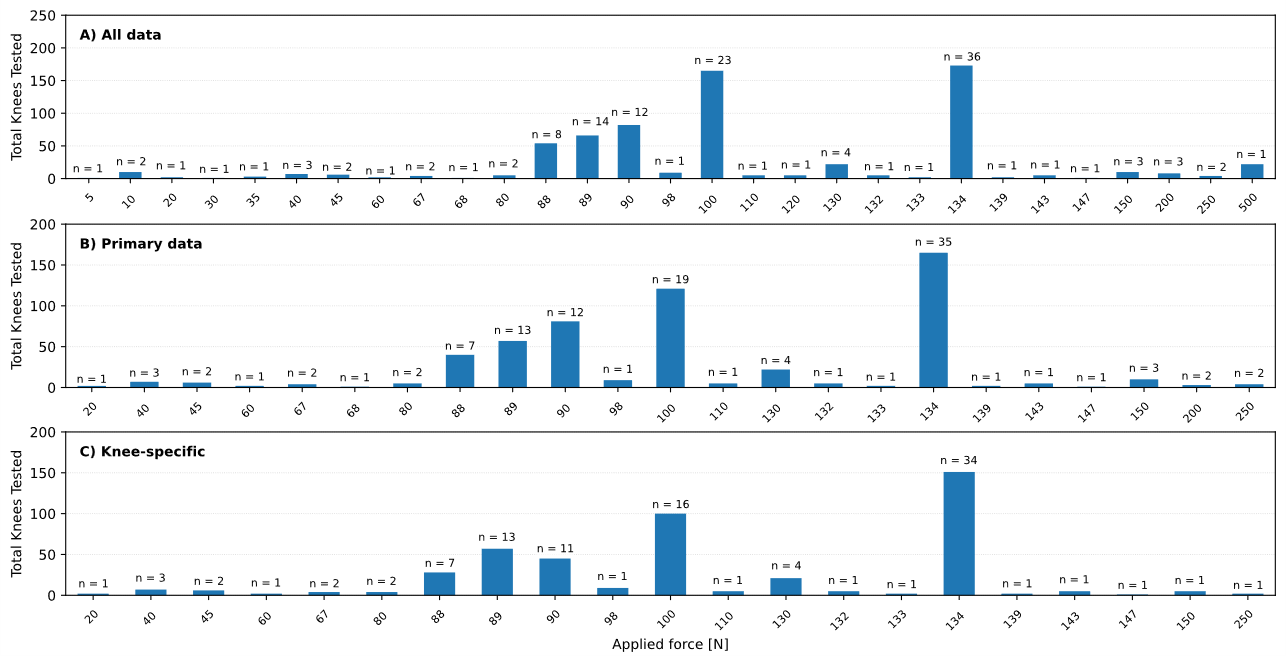


**Supplementary Figure 1.1.** Bar plot overview of applied **anterior force** in the summarised literature. A) All unique forces protocols applied, B) applied forces only filtered for primary laxity under single load protocols, and C) applied forces filtered for primary laxity under single load protocols and at least one of the defined knee flexion angles were included in the dataset. Annotation above each bar indicates the number of studies.


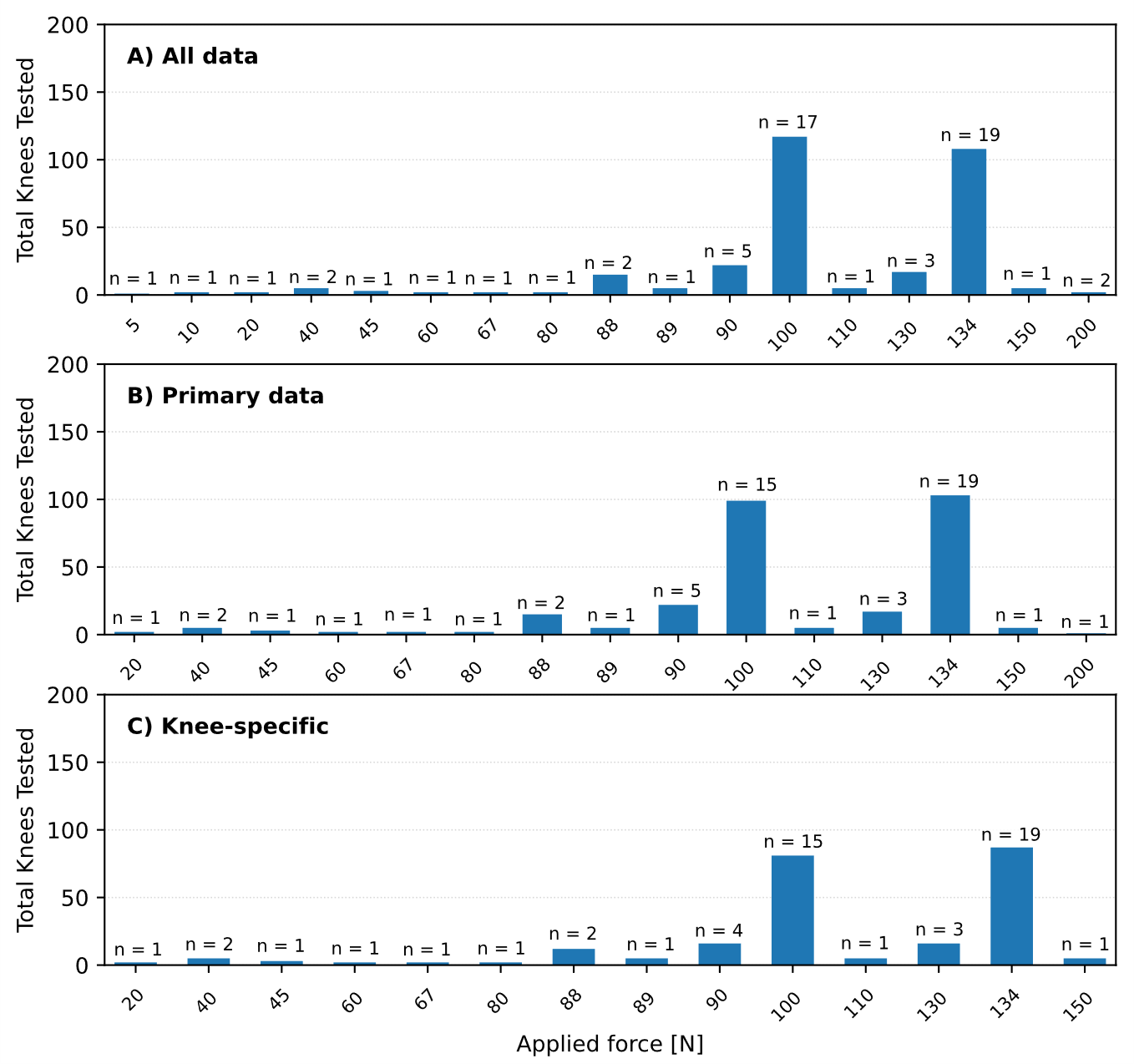
**Supplementary Figure 1.2.** Bar plot overview of applied **posterior force** in the summarised literature. A) All unique forces applied during both primary and secondary laxity testing, B) applied forces only filtered for primary laxity under single load protocols, and C) applied forces filtered for primary laxity under single load protocols and at least one of the defined knee flexion angles were included in the dataset. Annotation above each bar indicates the number of studies.


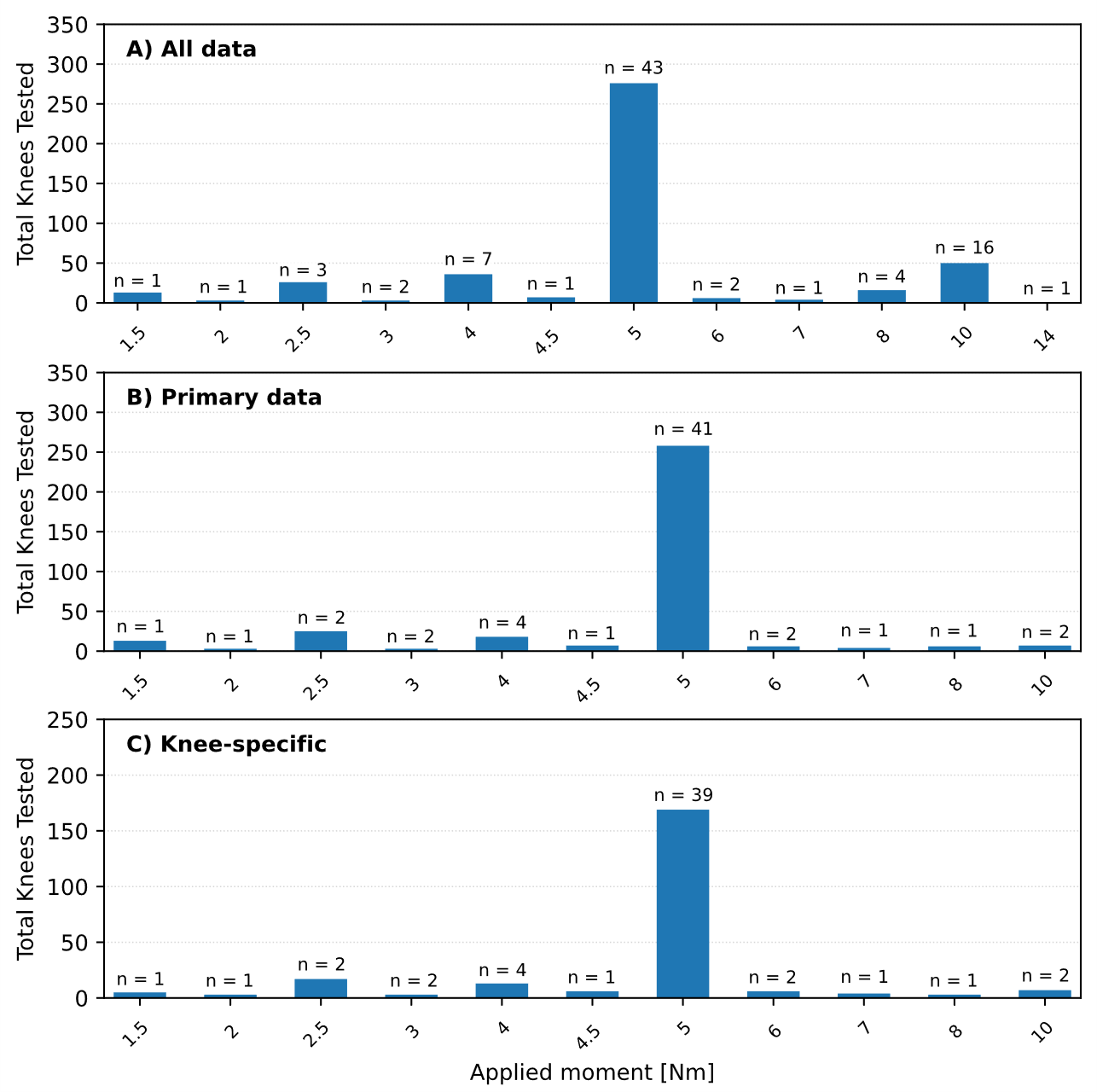


**Supplementary Figure 1.3.** Bar plot overview of applied **internal moments** in the summarised literature. A) All unique moments applied during both primary and secondary laxity testing, B) applied moments only filtered for primary laxity under single load protocols, and C) applied moments filtered for primary laxity under single load protocols and at least one of the defined knee flexion angles were included in the dataset. Annotation above each bar indicates the number of studies.


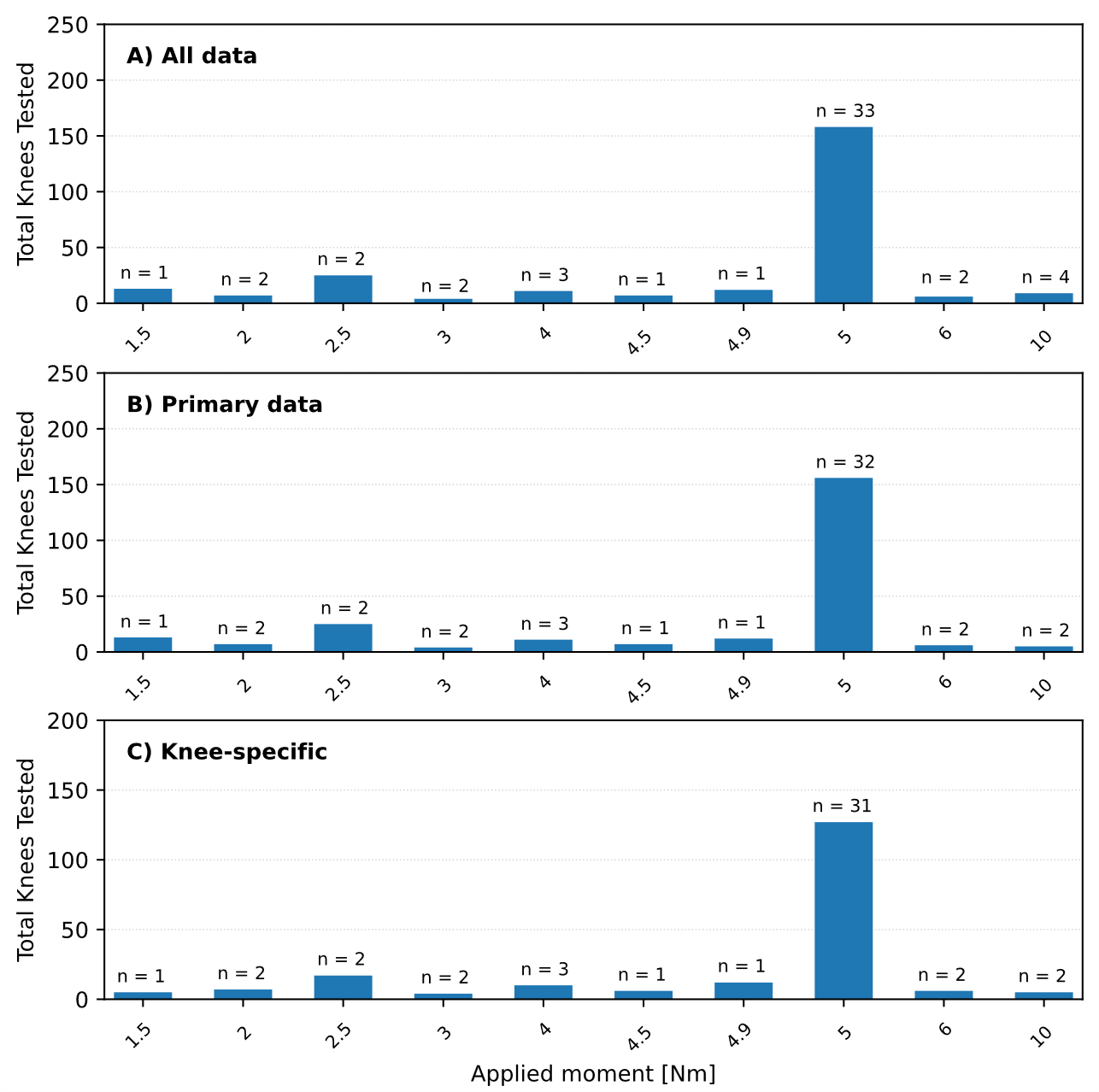


**Supplementary Figure 1.4.** Bar plot overview of applied **external moments** in the summarised literature. A) All unique moments applied during both primary and secondary laxity testing, B) applied moments only filtered for primary laxity under single load protocols, and C) applied moments filtered for primary laxity under single load protocols and at least one of the defined knee flexion angles were included in the dataset. Annotation above each bar indicates the number of studies.


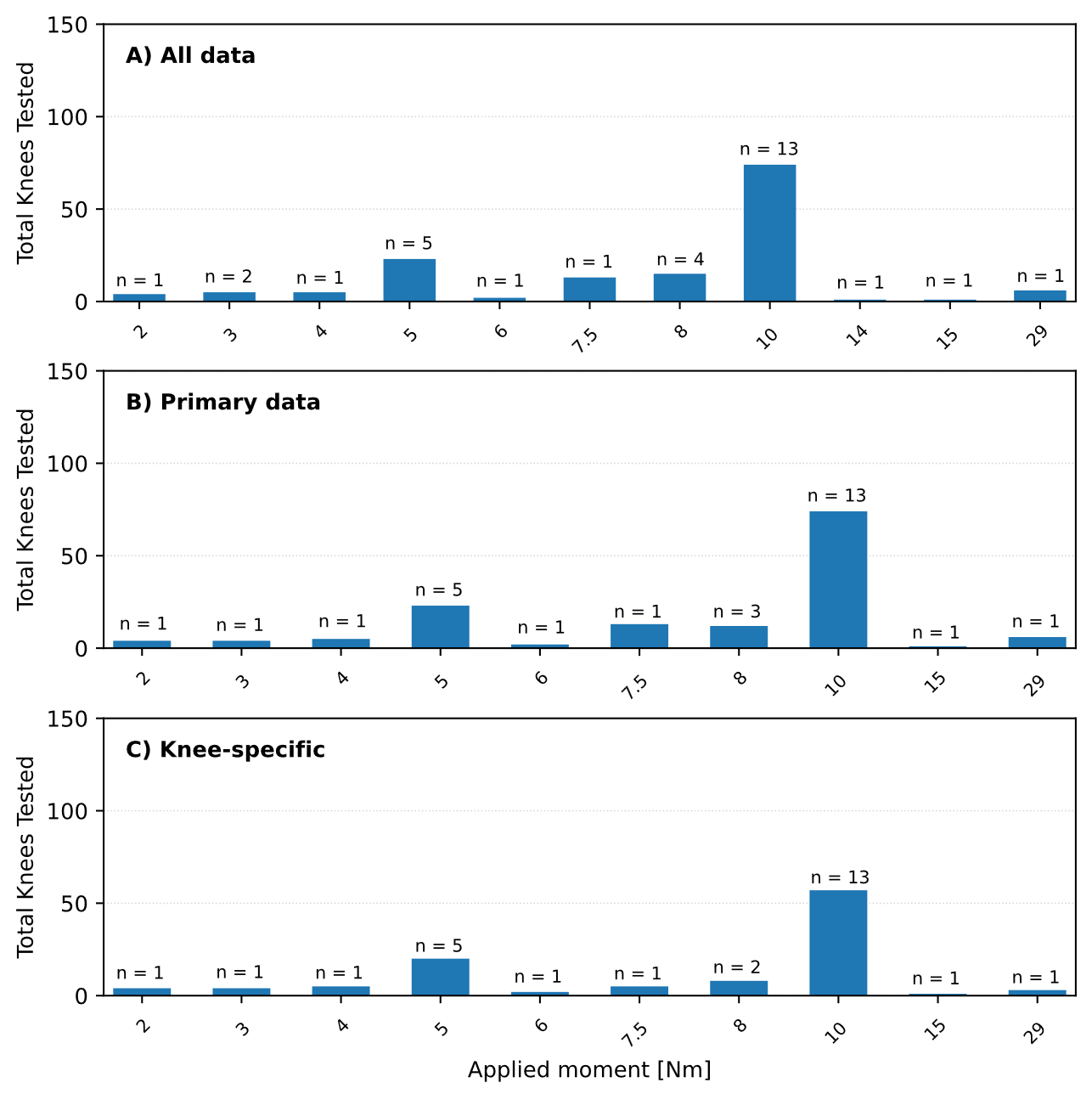


**Supplementary Figure 1.5.** Bar plot overview of applied **varus moments** in the summarised literature. A) All unique moments applied during both primary and secondary laxity testing, B) applied moments only filtered for primary laxity under single load protocols, and C) applied moments filtered for primary laxity under single load protocols and at least one of the defined knee flexion angles were included in the dataset. Annotation above each bar indicates the number of studies.


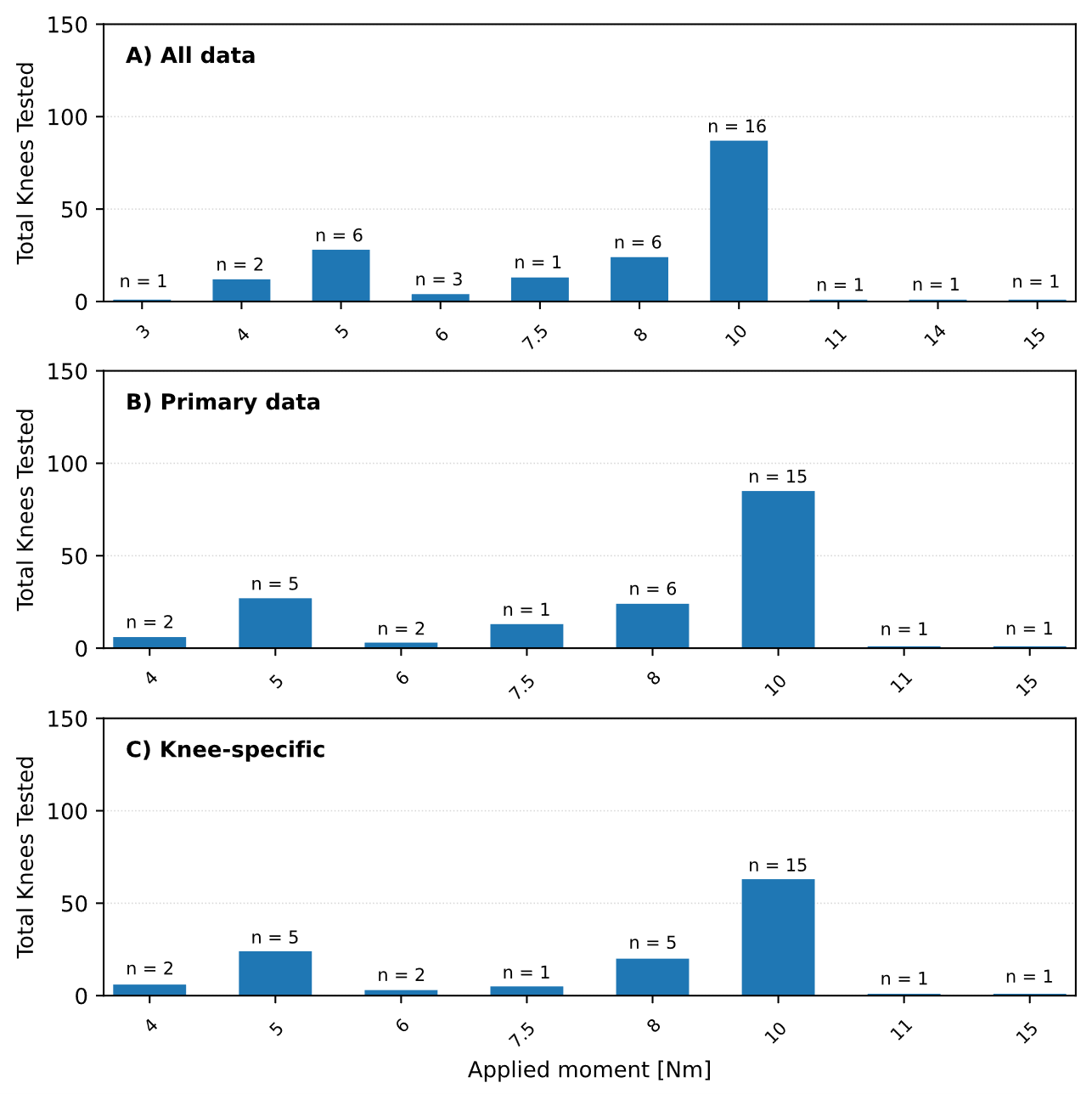


**Supplementary Figure 1.6.** Bar plot overview of applied **valgus moments** in the summarised literature. A) All unique moments applied during both primary and secondary laxity testing, B) applied moments only filtered for primary laxity under single load protocols, and C) applied moments filtered for primary laxity under single load protocols and at least one of the defined knee flexion angles were included in the dataset. Annotation above each bar indicates the number of studies.


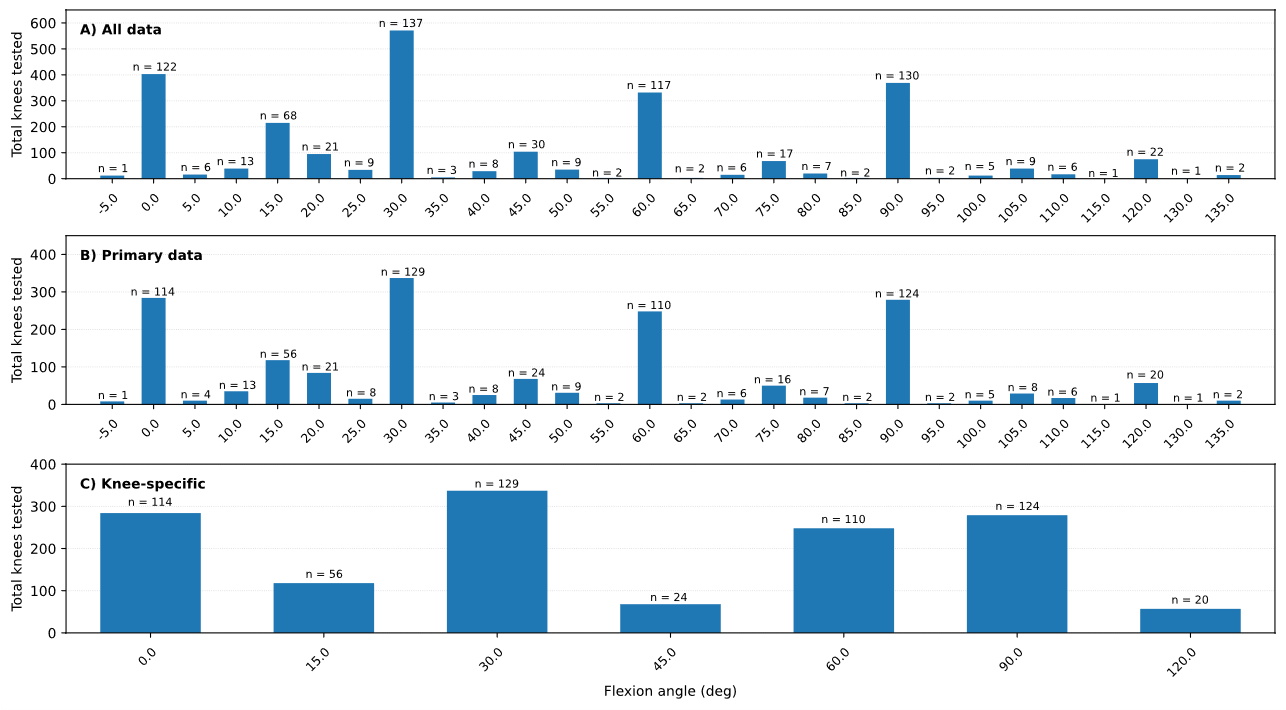


**Supplementary Figure 1.7.** Bar plot overview of used knee flexion angles in the summarised literature. A) All unique knee flexion angles reported during all different load protocols, B) knee flexion angles filtered for primary laxity under single load protocols, and C) specific knee flexion angles used in data reporting during grouping of knee laxity. Annotation above each bar indicates the number of studies.
